# A Preliminary DTI Tractography Study of Developmental Neuroplasticity 5–15 Years After Early Childhood Traumatic Brain Injury

**DOI:** 10.3389/fneur.2021.734055

**Published:** 2021-12-23

**Authors:** Elisabeth A. Wilde, Ilirjana Hyseni, Hannah M. Lindsey, Jessica Faber, James M. McHenry, Erin D. Bigler, Brian D. Biekman, Laura L. Hollowell, Stephen R. McCauley, Jill V. Hunter, Linda Ewing-Cobbs, Mary E. Aitken, Marianne MacLeod, Zili D. Chu, Linda J. Noble-Haeusslein, Harvey S. Levin

**Affiliations:** ^1^Department of Neurology, Traumatic Brain Injury and Concussion Center, University of Utah, Salt Lake City, UT, United States; ^2^H. Ben Taub Department of Physical Medicine and Rehabilitation, Baylor College of Medicine, Houston, TX, United States; ^3^Department of Neurology, Baylor College of Medicine, Houston, TX, United States; ^4^Department of Radiology, Baylor College of Medicine, Houston, TX, United States; ^5^Department of Pediatrics, Baylor College of Medicine, Houston, TX, United States; ^6^Department of Psychology, Brigham Young University, Provo, UT, United States; ^7^Department of Psychology, University of Minnesota Twin Cities, Minneapolis, MN, United States; ^8^Department of Pediatrics, University of Texas Health Science Center at Houston, Houston, TX, United States; ^9^Department of Pediatric Radiology, Texas Children's Hospital, Houston, TX, United States; ^10^Department of Pediatrics, University of Arkansas for Medical Sciences, Little Rock, AR, United States; ^11^Departments of Psychology and Neurology, University of Texas at Austin, Austin, TX, United States

**Keywords:** pediatric traumatic brain injury, diffusion tensor imaging, tractography, brain development, neuroplasticity, structural neuroimaging

## Abstract

Plasticity is often implicated as a reparative mechanism when addressing structural and functional brain development in young children following traumatic brain injury (TBI); however, conventional imaging methods may not capture the complexities of post-trauma development. The present study examined the cingulum bundles and perforant pathways using diffusion tensor imaging (DTI) in 21 children and adolescents (ages 10–18 years) 5–15 years after sustaining early childhood TBI in comparison with 19 demographically-matched typically-developing children. Verbal memory and executive functioning were also evaluated and analyzed in relation to DTI metrics. Beyond the expected direction of quantitative DTI metrics in the TBI group, we also found qualitative differences in the streamline density of both pathways generated from DTI tractography in over half of those with early TBI. These children exhibited hypertrophic cingulum bundles relative to the comparison group, and the number of tract streamlines negatively correlated with age at injury, particularly in the late-developing anterior regions of the cingulum; however, streamline density did not relate to executive functioning. Although streamline density of the perforant pathway was not related to age at injury, streamline density of the left perforant pathway was significantly and positively related to verbal memory scores in those with TBI, and a moderate effect size was found in the right hemisphere. DTI tractography may provide insight into developmental plasticity in children post-injury. While traditional DTI metrics demonstrate expected relations to cognitive performance in group-based analyses, altered growth is reflected in the white matter structures themselves in some children several years post-injury. Whether this plasticity is adaptive or maladaptive, and whether the alterations are structure-specific, warrants further investigation.

## Introduction

The concepts of increased vulnerability and neuroplasticity have been used to understand recovery from early traumatic brain injury (TBI), yet the interaction of the timing of brain insult with developmental factors that influence recovery remains unclear (1, 2). Some have postulated early childhood TBI may critically disrupt subsequent synaptic organization and modify neural network formation, whereas later TBI may have more localized effects (3–5). In contrast, some children with acquired injury early in life have exhibited remarkable resiliency, suggesting a capacity for reorganization may also be present (6).

Diffuse axonal injury (DAI) is a consequence of wide spread deformation to white matter (WM) fiber systems that often results from the biomechanics of TBI (7, 8). DAI may affect WM integrity by disrupting neural networks (9), and diffusion tensor imaging (DTI), which is more sensitive to structural alteration in pediatric TBI than conventional MRI (10), is ideally suited to explore potential WM changes related to TBI and age at the time of injury. DTI measures the diffusion of water molecules in brain tissue to interrogate WM integrity or organization *via* several quantitative diffusion metrics, including fractional anisotropy (FA), a measure of the extent to which diffusion is restricted, and mean diffusivity (MD), a measure of the average rate of diffusion within a voxel (10). Axial (AD) and radial diffusivity (RD) are directional measures of the rate of diffusion that may be sensitive to changes in axonal integrity (11) and myelination (12), respectively. DTI tractography is a quantitative post-processing method by which discrete tract streamlines that model WM pathways are generated (13).

There is limited research into the effects of early TBI on WM integrity several years post-injury; however, a large study of children and adolescents with moderate-to-severe TBI found disruptions to the integrity of various WM tracts at the acute/subacute, postacute, and chronic (6–26 months) post-injury periods (14). Specifically, significantly lower FA and higher MD was observed across tracts in those with TBI, relative to controls, at all three post-injury periods. *Post-hoc* analysis revealed higher RD at each post-injury period, while AD was observed to be lower acutely but higher at later post-injury periods across the majority of pathways. This study also found that, while injury severity was a significant contributor to the extent of WM alterations early on, the amount of variance in diffusion metrics explained by injury severity decreased over time.

**Figure 1:**
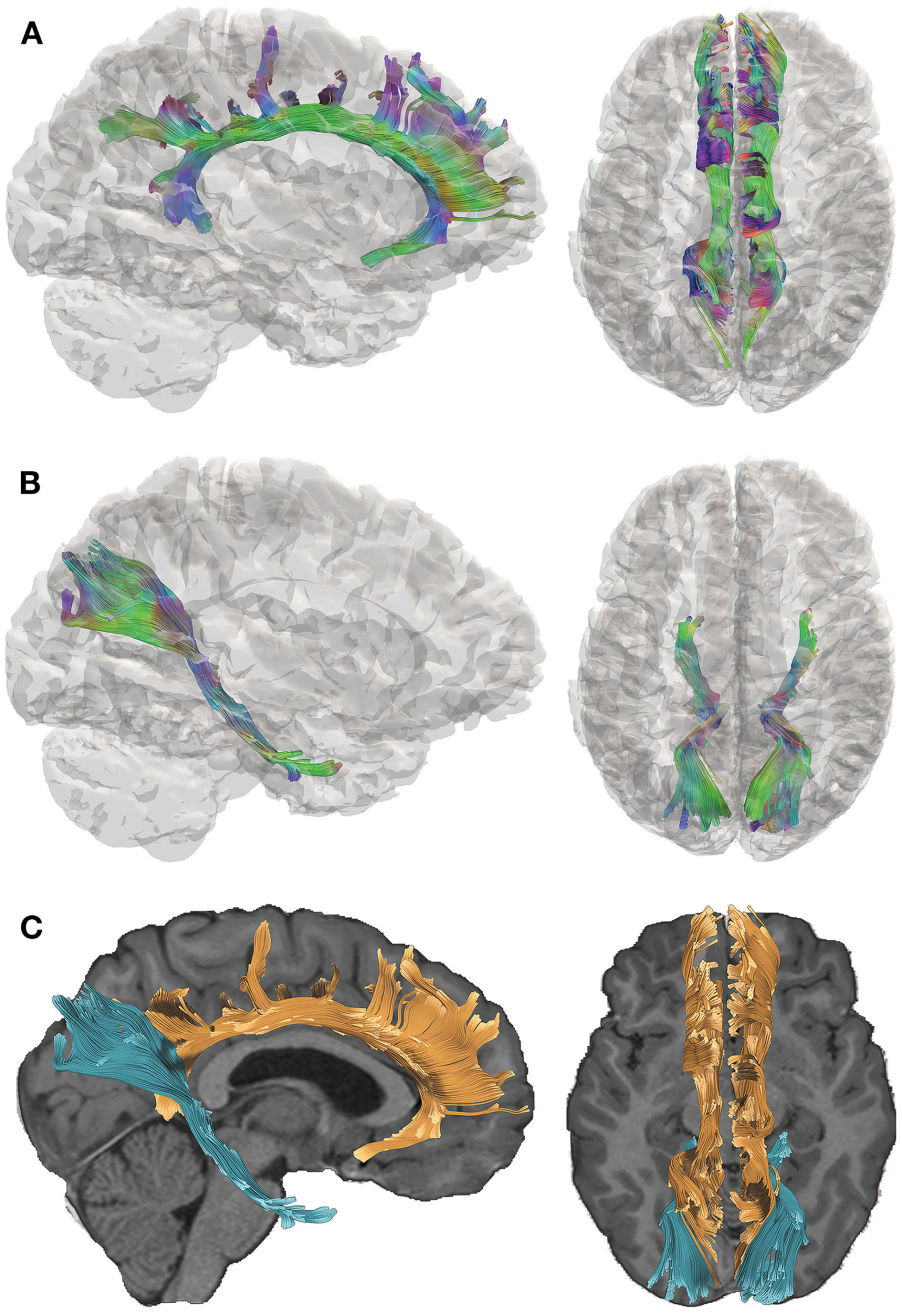
Tractography of the cingulum bundle (CB) and perforant pathway (PP). Directional color maps of the right CB **(A)** and PP **(B)** are shown on the sagittal (left) and axial (right) planes, overlaid by a 3D cortical isosurface. The colors represent the 3D fiber rendering direction; red, left-to-right (along the *x*-axis); green, anterior-to-posterior (along the *y*-axis); and blue, superior-to-inferior (along the *z*-axis). The bottom panel **(C)** demonstrates the spatial relationship of the CB (orange) and PP (blue), which are overlaid on the subject's T1-weighted image. The right side of the brain is depicted in the left side of the screen in the axial views.

We acquired DTI data 5–15 years after complicated mild, moderate, or severe TBI sustained during young childhood and utilized quantitative tractography to interrogate two pathways that are implicated in the cognitive consequences of TBI (15). The late-developing cingulum bundle (CB; Figure 1A) connects the prefrontal, posterior, and limbic regions and has been associated with cognitive control and executive functioning. The perforant pathway (PP; Figure 1B) projects to the entorhinal cortex and hippocampal formation and is linked to memory functioning (16). The spatial relationship of the CB relative to the PP is demonstrated in Figure 1C. Furthermore, the CB and PP are particularly vulnerable to TBI, as they connect frontal and temporal regions of the brain that are susceptible to damage due to their situation within the anterior and cranial fossa of the skull (17). Consistent with our focus on the CB and PP, we selected tests of episodic memory and executive function that are well-validated in TBI populations (18–20) for this study. Episodic memory was assessed using the California Verbal Learning Test [CVLT (21, 22)], which is a measure of list learning and immediate and delayed verbal recall. Executive functioning was assessed using the Delis-Kaplan Executive Functioning System [D-KEFS; (23)] Color-Word Interference Test (CWIT), which is used to measure inhibition of over-learned responses through performance across color naming (CN), word reading (WR), inhibition (IN), and inhibition/switching (IS) conditions. In this exploratory investigation, we investigated whether early TBI would adversely affect later structural development and functionality of these pathways.

## Materials and Methods

The data used in the present study were part of a research program on pediatric TBI that was conducted at Baylor College of Medicine and the University of Arkansas for Medical Sciences. This study was approved by the Institutional Review Board at all participating sites. Written informed consent was obtained from all guardians of participants, as well as verbal assent from child participants, prior to enrollment, prior the acquisition of MRI data, and prior to the neuropsychological assessment.

### Participants

The present study included 21 patients (29% female) aged 10–18 years at the time of the evaluation, who sustained a TBI between the ages of 1–8 years (mean = 4.10 ± 2.02). Although we had designed the study to focus on children injured at age 5 years or younger, difficulty in recruiting children who met the eligibility criteria (see below) necessitated that we expand the upper age at injury range to 8 years. Capability of undergoing unsedated DTI was also a consideration in specifying the youngest current age of children selected for this follow-up study, which was 10 years in the TBI group (Table 1). Patients were enrolled in the study between 5.8 and 14.7 years (mean = 9.51 ± 2.73) post-injury. Based on initial Glasgow Coma Scale [GCS (24)] scores obtained at the scene by first responders, TBI severity was classified as mild (GCS = 13–15) complicated by intracranial findings on initial CT, moderate (GCS = 9–12), and severe (GCS = 3–8). Initial GCS scores of the present sample ranged from 3 to 15 (mean = 7.86 ± 3.82), where complicated mild, moderate, and severe injuries were sustained by 14%, 19%, and 67% of TBI patients, respectively. In addition to other injury characteristics, Table 2 lists the pathology identified by acute clinical imaging for each TBI patient; the type of brain injury was heterogeneous, including 11 cases of hemorrhage or hematoma, two patients with contusions, and a single case diagnosed as DAI. The most common mechanisms of injury were falls (33%) and motor vehicle accidents (24% passenger, 10% pedestrian), although other injury mechanisms included blunt-force trauma (14%), recreational vehicle accidents (9.5%), and sport- or play-related injuries (9.5%).

**Table 1:** Demographic characteristics of the present sample.

	**TBI (*****n*** **= 21)**		**TDC (*****n*** **= 19)**		**Inferential Statistics**		
	***N***	**%**	***n***	**%**	**χ^2^**	***df***	***p***
Sex					0.04	1	0.836
Male	15	71.4	13	68.4			
Female	6	28.6	6	31.6			
Ethnicity					1.15	2	0.851
African-American	0	0.0	1	5.3			
Caucasian	16	76.2	14	73.7			
Hispanic/Latin-American	5	23.8	4	21.1			
Dominant Hand					0.02	1	0.894
Right	18	85.7	16	84.2			
Left	3	14.3	3	15.8			
	**M ± SD**	**Range**	**M ± SD**	**Range**	***t***	***df***	***P***
Age at Evaluation	13.57 ± 2.38	10 to 18	13.58 ± 2.34	9 to 17	0.01	38	0.992
SES^a^	−0.16 ± 1.13	−2.61 to 1.44	0.17 ± 0.84	−0.97 to 1.56	0.99	35	0.328

*TBI, traumatic brain injury; TDC, typically-developing child; SES, socioeconomic status*.

a *SES was determined using the Socioeconomic Composite Index^14^ and is reported in z-scores (mean = 0.00 ± 1.00), which were standardized from the present sample*.

**Table 2:** Injury details for each child and adolescent with traumatic brain injury.

**Patient**	**Age at injury**	**Years since injury**	**Age at evaluation**	**Mechanism of injury**	**Initial GCS**	**Injury severity classification**	**Initial CT results**		
							**Primary injury**	**Location**	**Hemisphere**
1	5	8.53	13	Sports/Play	7	Severe	EDH	Frontal, Parietal	Left
2	4	9.77	13	MVA	11	Moderate	SAH	Lateral and Fourth Ventricles	Bilateral
3	2	13.13	15	Fall	8	Severe	SAH	Temporal	Left
4	5	8.47	13	Fall	5	Severe	EDH	Frontal, Parietal	Right
5	4	8.31	12	BFT	10	Moderate	SDH	Parietal	Right
6	5	6.45	11	MVA	3	Severe	Depressed SF	Frontal	Right
7	5	6.19	11	RVA	7	Severe	SDH	Posterior Fossa	Left
8	8	8.94	16	RVA	3	Severe	SAH	Not specified	Left
9	1	14.69	15	Pedestrian	8	Severe	Basilar SF	Occipital	Bilateral
10	8	5.85	13	MVA	3	Severe	DAI	Frontal, Temporal, Basal Ganglia	Bilateral
11	8	5.79	13	Sports/Play	12	Moderate	Contusion	Occipital	Bilateral
12	2	9.77	11	Pedestrian	8	Severe	Basilar SF	Temporal, Parietal	Bilateral
13	4	8.66	12	BFT	6	Severe	Depressed SF	Parietal, Occipital	Left
14	2	9.28	11	Fall	13	Complicated Mild	IVH	Choroid Plexus, Lateral Ventricle	Right
15	2	9.08	11	Fall	8	Severe	Basilar SF	Cranial fossa	Left
16	3	10.06	13	Fall	8	Severe	Basilar SF	Occipital	Midline
17	3	11.92	14	Fall	15	Complicated Mild	IVH	Occipital horns of Lateral Ventricle	Bilateral
18	4	13.94	17	MVA	3	Severe	Contusion	Temporal	Right
19	3	14.55	17	BFT	9	Moderate	CBH	Not specified	Right
20	3	7.30	10	MVA	3	Severe	SF	Temporal	Right
21	5	9.10	14	Fall	15	Complicated Mild	PCH	Parietal	Left

*GCS, Glasgow Coma Scale score; CT, computed tomography; MVA, motor vehicle accident (passenger); BFT, blunt-force trauma; RVA, recreational vehicle accident; EDH, epidural hematoma; SAH, subarachnoid hemorrhage; SDH, subdural hematoma; SF, skull fracture; DAI, diffuse axonal injury; IVH, intraventricular hemorrhage; CBH, cerebellar hematoma; PCH, parenchymal hematoma*.

The comparison group included 19 typically-developing children (TDC; 32% female), aged 9–17 years (mean = 13.58 ± 2.34). The groups were demographically-comparable on age at evaluation, sex, ethnicity, handedness, and socioeconomic status (SES; Table 1). SES was determined using the Socioeconomic Composite Index (SCI), according to the guidelines described by Yeates et al. (25). Exclusion criteria for both groups included contradictions to MRI, history of child abuse, and prior diagnosis of psychotic or neurologic disorders. Exclusion criteria specific to the TDC group included prior head injury that required hospitalization, whereas children were excluded from the TBI group if any head injury that required hospitalization occurred prior to or following the index injury.

### Neuroimaging Protocol

Participants underwent MRI without sedation on Philips 3T Achieva scanners (Philips, Cleveland, OH) at Texas Children's Hospital in Houston (12 TBI, 11 TDC) or at the University of Arkansas in Little Rock (9 TBI, 8 TDC) using comparable platforms and software. Regular quality assurance testing was performed on both scanners, and both were consistently found to be within specification throughout the duration of the study. No systematic differences in DTI metrics derived between sites for TBI or comparison participants were observed (Supplementary Table 1). Additionally, there was no relationship between study site and the number of TBI or TDC participants recruited, χ^2^(1) = 0.002, *p* = 0.962.

**Figure 2:**
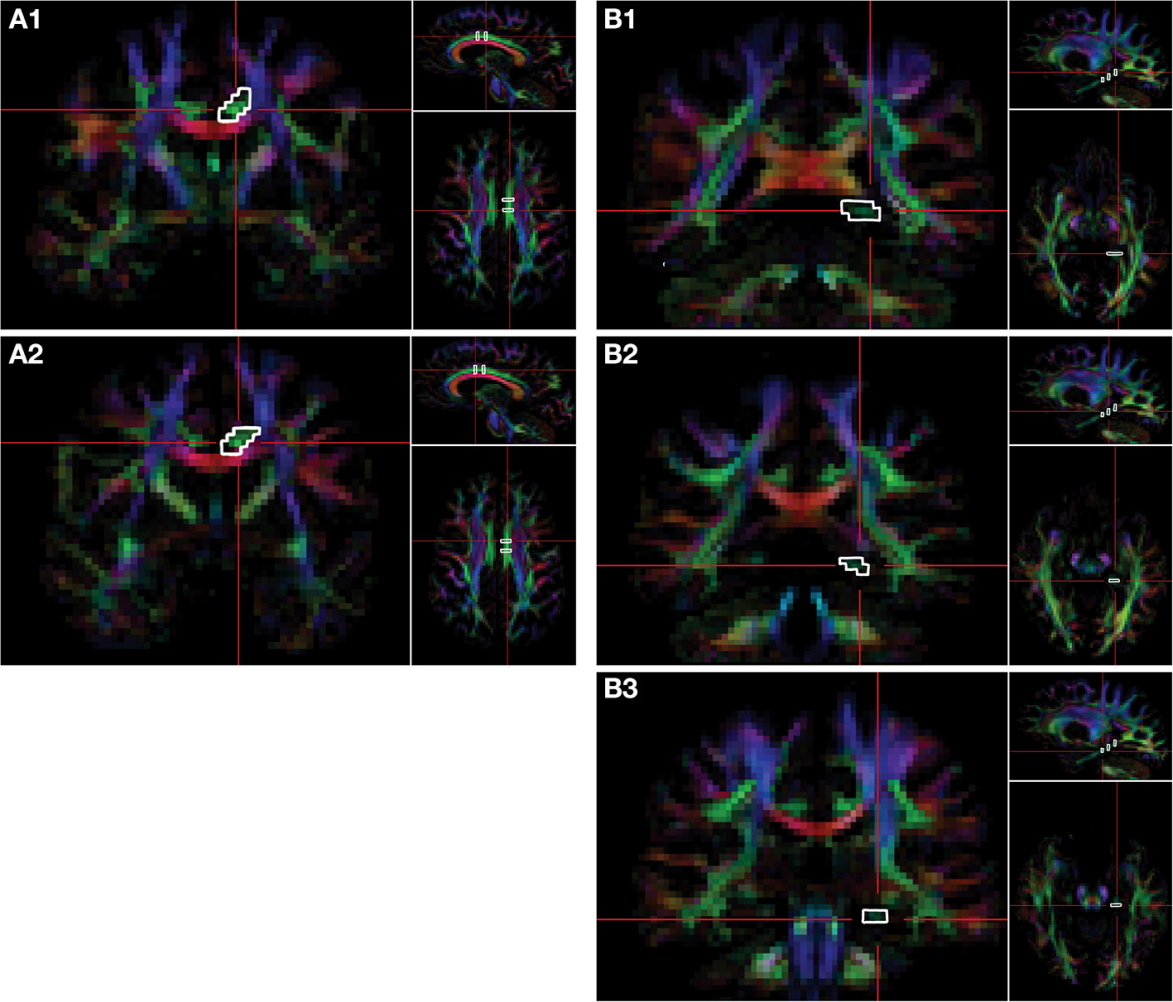
Delination of regions-of-interest for the CB and PP on the FA color map. Fiber tracts of cingulum bundle (CB) and perforant pathway (PP) are isolated *via* regions of interest (ROIs). Two ROIs of the CB are selected on the coronal plane. The first ROI of the CB **(A1)** is selected at the level of the fornix body four slices posterior to the second ROI **(A2)**, which is selected at the level of the fornix column. Three ROIs are selected on the coronal plane for the PP. The first ROI is selected on the most anterior slice of the splenium of the corpus callosum **(B1)**, the second ROI of PP is selected three slices anterior to the first **(B2)**, and the third ROI is selected six slices anterior to the first ROI **(B3)**. The right side of the brain is depicted in the left side of the screen. The colors represent the 3D fiber rendering direction; red, left-to-right (along the *x*-axis); green, anterior-to-posterior (along the *y*-axis); and blue, superior-to-inferior (along the *z*-axis). FA, fractional anisotropy.

DTI acquisition involved transverse multi-slice spin echo, single shot, echoplanar imaging (EPI) sequences (TR/TE = 7,305/51 ms, slice thickness/gap = 2/2 mm, matrix = 112 × 110 mm, voxel size = 1.75 × 1.75 × 2.0 mm), with diffusivities measured along 32 directions using a low/high *b*-value of 0/1,000 s/mm^2^. To achieve higher signal-to-noise ratio (SNR) and improve the reproducibility of the generated tract streamlines, two DTI acquisitions were acquired and later averaged using the Philips diffusion affine registration program (26). During the averaging process, any eddy current distortion and head motion artifact were corrected. Philips fiber tracking 4.1V3 Beta 2 software [Philips, Best, The Netherlands (27)] was used to quantify and extract FA, MD, AD, and RD maps and perform the fiber tractography using a deterministic algorithm based on the fiber assignment by continuous tracking (FACT) method (28) with Runge-Kutta interpolation. Tracking was terminated in voxels when FA fell below 0.2 or if the angular threshold of 7° was met. Quantitative tractography was performed manually using multiple regions of interest (ROIs) for the CB and PP in each hemisphere, utilizing protocols previously published by our group and others (15, 16, 29–31), which yield a point score for each identifiable streamline within a ROI. Details of the ROI selection and tract isolation procedure are provided in Figure 2. It is important to note that the number of streamline points (i.e., streamline density) does not reflect the true underlying fiber density but is dependent on the fiber tracking algorithm used (32).

To ensure inter-operator reliability of DTI protocols, two experienced raters independently analyzed a subset of the study data (6 TBI and 6 TDC), and all measures were analyzed twice by the same rater. Shrout-Fleiss reliability statistics were used to provide intra-class correlation coefficients (ICCs) as a measure to establish intra- and inter-rater reliability; ICCs for each measurement were above 0.97. All DTI analysts were masked to the participants' performance on neuropsychological testing.

### Cognitive Assessment

The following assessments were administered as part of a larger battery of neuropsychological tests. All participants completed the cognitive assessment on the same day as they underwent neuroimaging. All of the test administrators were masked to the results of DTI analyses.

Verbal learning and memory was assessed using the standard five-trial version of the CVLT–Children's version [CVLT-C (21)] for children aged 5–16 years, and the CVLT–Second edition [CVLT-II (22)] for participants aged 17–18 years. List recall performance across the five learning trials (Trials 1–5), short-delay free recall (SDFR)–requiring recall of the word list following a brief delay after the initial five learning trials–and long-delay free (LDFR)–requiring recall of the word list after a 20-min delay–were analyzed. Raw scores from the CVLT were standardized using demographically-corrected normative data provided in test administration manual, and the resulting standardized scores [T-scores (mean = 50 ± 10) for Trials 1–5; *z*-scores (mean = 0.0 ± 1.0) for SDFR and LDFR] were used in all statistical analyses.

The D-KEFS CWIT was used to assess executive function, where the first two conditions (CN, WR) assess baseline competency in identifying the color of patches and reading color words in black ink. The third condition (IN) requires the examinee to name the color of ink that color words are printed in while inhibiting naming the color word itself. The fourth condition (IS) measures cognitive flexibility and the ability to inhibit an overlearned response (33). Specifically, this condition requires the examinee to switch between naming the color of ink that color words are printed in and reading color words, which are printed in ink of a different color (e.g., “red” printed in green ink). Raw scores from the CWIT were standardized using demographically-corrected normative data provided in test administration manual, and the resulting scaled scores (mean = 10 ± 3) were used in all statistical analyses.

### Statistical Analysis

All continuous demographic variables were examined for normality and homogeneity of variance prior to between-group comparisons. Independent *t*-tests were used to assess group differences in age at evaluation and SES (i.e., SCI *z*-score). Distributions of sex, handedness, and ethnicity between groups were compared using Pearson's chi-square or Fisher's exact tests, appropriately. Within-group correlational analyses were also conducted to determine if relationships existed between sex (point-biserial *r*), age at evaluation, and SES (Pearson's *r*) with all cognitive and DTI variables.

Due to violations of homogeneity of variance for a number of the cognitive testing and DTI variables, age at evaluation, sex, and SES were not included as covariates in our models; rather, two-tailed Welch's *t*-tests (34) were used to compare performance on cognitive measures (CVLT: Trials 1–5, SDFR, and LDFR; CWIT: CN, WR, IN, and IS) and DTI parameters (FA, MD, and number of streamline points) of the CB and PP between TBI and TDC groups. All comparisons included the calculation of 95% confidence intervals (CIs) and Hedges's *g* as a measure of effect size (35), where |*g|* ≥ 0.20, 0.50, and 0.80 are considered small, moderate, and large effects, respectively (36).

Partial correlation analyses were conducted within the TBI group to explore the relation between DTI parameters (streamline points, FA, MD) extracted from the CB and PP and cognitive functioning. Partial correlations were also used to explore the relationship between integrity of the CB and PP and age at injury. The effects of age at evaluation, sex, and SES were statistically controlled for in all correlational analyses, and squared semipartial correlations are included as estimates of effect size, where $r_{\text{sp}}^{2}$ ≥ 0.01, 0.09, and 0.25 are considered small, moderate, and large effects, respectively (36).

Supplementary analyses were conducted to explore differences in measures of directional diffusivity (AD, RD) extracted from the CB and PP between groups using Welch's *t*-tests, as well as relationships between AD and RD with cognitive functioning and age at injury using partial correlations. As with the primary analyses, the effects of age at evaluation, sex, and SES were statistically controlled for in all correlational analyses, and Hedges's *g* and squared semipartial correlations are included as estimates of effect size for between-group comparisons and partial correlations, respectively.

Given the exploratory nature of the study, formal correction for multiple comparisons was not performed for the primary or supplementary analyses; exact *p*-values are reported for direct evaluation of statistical significance (33). All statistical analyses were performed using Stata version 16.0 (StataCorp LLC, College Station, TX).

## Results

### Group-Level Comparisons

No significant group differences were observed for demographic variables (Table 1). Within the TDC group, sex was correlated with FA in the right PP (*r*_pb_ = −0.47, *p* = 0.042) and with the number of streamline points in the right CB (*r*_pb_ = −0.51, *p* = 0.027); however, there were no correlations between sex and DTI metrics for any ROI in the TBI group. No relationship was found for sex with any cognitive variable in the TDC group; however, sex-related differences were apparent in the TBI group for performance on the CN (*r*_pb_ = 0.46, *p* = 0.037), WR (*r*_pb_ = 0.46, *p* = 0.038), and IN (*r*_pb_ = 0.47, *p* = 0.030) conditions of the CWIT. Age at evaluation was related to WR performance within the TBI group (*r* = −0.44, *p* = 0.047); however, no other relationships were found for age at evaluation with any cognitive or DTI variables in either group.

Significant differences were found between the TBI and TDC groups for several cognitive variables (Table 3). The TBI group performed more poorly than the TDC group on WR, IN, and IS conditions of the CWIT, and a moderate effect size was found for poorer CN performance in the TBI group. Similarly, the TBI group scored lower on Trials 1–5 and SDFR of the CVLT, and a moderate effect size was found for poorer LDFR performance in the TBI group.

**Table 3:** Between-group differences in cognitive performance and diffusion metrics.

	**TBI (*****n*** **= 21)**		**TDC (*****n*** **= 19)**		***t***	***df* ^a^**	***p***	**95% CI**		***g***
	***M***	***SD***	***M***	***SD***				***LL***	***UL***	
**Cognitive performance**										
CVLT										
Trials 1–5	**43.19**	**13.69**	**54.21**	**8.96**	**3.04**	**36.31**	**0.004**	**3.67**	**18.37**	**-0.92**
SDFR	**−0.69**	**1.44**	**0.16**	**0.93**	**2.24**	**36.09**	**0.031**	**0.08**	**1.62**	**−0.68**
LDFR	−0.29	1.48	0.42	0.99	1.79	36.72	0.082	**–**0.09	1.51	**–**0.54
CWIT										
CN	8.43	3.78	10.11	2.16	1.74	33.64	0.090	**–**0.28	3.63	**–**0.53
WR	**7.95**	**3.76**	**10.26**	**3.02**	**2.15**	**39.39**	**0.038**	**0.14**	**4.48**	**−0.66**
IN	**7.71**	**3.49**	**10.37**	**2.77**	**2.67**	**39.28**	**0.011**	**0.65**	**4.66**	**−0.82**
IS	**7.55**	**3.58**	**10.32**	**2.31**	**2.88**	**34.18**	**0.007**	**0.82**	**4.72**	**−0.89**
**Diffusion Metrics**										
Streamline Points^b^										
CB Right	708.83	416.08	607.83	340.40	0.84	39.57	0.404	**–**343.12	141.13	0.26
CB Left	919.99	484.32	817.97	424.38	0.71	39.95	0.482	**–**392.46	188.41	0.22
PP Right	152.85	160.75	196.65	123.80	0.97	38.94	0.338	−47.50	135.10	**–**0.30
PP Left	135.99	97.28	139.48	86.74	0.12	39.99	0.905	**–**55.32	62.30	**–**0.04
FA										
CB Right	0.38	0.04	0.40	0.03	1.57	33.79	0.127	**–**0.01	0.04	**–**0.47
CB Left	0.41	0.05	0.43	0.03	1.43	35.66	0.162	**–**0.01	0.04	**–**0.43
PP Right	0.32	0.04	0.34	0.02	1.82	26.45	0.081	0.00	0.04	**–**0.54
PP Left	0.32	0.04	0.33	0.02	1.11	35.33	0.274	**–**0.01	0.03	**–**0.34
MD										
CB Right	0.77	0.03	0.76	0.02	1.79	35.66	0.082	**–**0.03	0.00	0.54
CB Left	**0.76**	**0.04**	**0.74**	**0.02**	**2.19**	**34.09**	**0.036**	**−0.04**	**0.00**	**0.66**
PP Right	0.82	0.06	0.81	0.03	1.05	32.37	0.299	**–**0.04	0.01	0.93
PP Left	0.82	0.06	0.80	0.04	0.84	35.67	0.404	**–**0.05	0.02	0.26

*Bold values indicate statistical significance (p < 0.05) without correction for multiple comparisons. Negative values of Hedges's g indicate lower values in the TBI group relative to the TDC group. Hedges's g-values are interpreted as small, medium, and large effect sizes when |g| ≥ 0.20, 0.50, and 0.80, respectively*.

*TBI, traumatic brain injury; TDC, typically-developing child; CVLT, California Verbal Learning Test; SDFR, short-delay free recall; LDFR, long-delay free recall; CWIT, Color-Word Interference Test; CN, color naming; WR, word reading; IN, inhibition; IS, inhibition/switching; CB, cingulum bundle; PP, perforant pathway; FA, fractional anisotropy; MD, mean diffusivity*.

a *Welch's approximation was applied to the degrees of freedom*.

b *Mean, standard deviation, and confidence intervals for the number of streamline points in each ROI are expressed in 1/1,000 units*.

With the exception of increased MD in the left CB of the TBI group, relative to the TDC group, no significant differences in the number of streamline points, FA, or MD were found between groups for either ROI (Table 3). However, moderate to large effect sizes were found for decreased FA in the right PP, increased MD in the right CB, and increased MD in the right PP of the TBI group, relative to the TDC group.

Supplementary analyses revealed significantly higher RD in the left CB and a moderate effect size for higher RD in the right CB of those with TBI relative to those in the TDC group (Supplementary Table 2). No group differences were found in AD of either pathway.

### Relation of DTI Parameters to Cognitive Performance and Age at Injury in TBI

Within the TBI group, increased FA of the left PP was related to better performance on the IN condition of the CWIT. No other significant relationships were seen between streamline points, FA, or MD of the CB or PP with performance on CWIT (Table 4).

**Table 4:** Partial correlations between performance on the Color-Word Interference Test (CWIT) and diffusion parameters.

	**Color naming**			**Word reading**			**Inhibition**			**Inhibition/switching**		
	***r*_p_**	***p***	$r_{sp}^{2}$	***r*_p_**	***p***	$r_{sp}^{2}$	***r*_p_**	***p***	$r_{sp}^{2}$	***r*_p_**	***p***	$r_{sp}^{2}$
Streamline points												
CB Right	−0.36	0.172	0.08	−0.11	0.689	0.01	−0.27	0.313	0.04	−0.44	0.099	0.13
CB Left	−0.13	0.633	0.01	0.23	0.398	0.03	−0.02	0.956	0.00	−0.16	0.572	0.02
PP Right	−0.09	0.748	0.00	−0.08	0.762	0.00	−0.05	0.863	0.00	−0.15	0.595	0.01
PP Left	0.38	0.149	0.09	0.10	0.704	0.01	0.22	0.409	0.03	0.25	0.376	0.04
FA												
CB Right	0.32	0.234	0.06	0.38	0.148	0.09	0.22	0.411	0.03	−0.05	0.857	0.00
CB Left	0.29	0.281	0.05	0.45	0.084	0.12	0.20	0.459	0.02	0.03	0.920	0.00
PP Right	0.27	0.320	0.04	0.22	0.423	0.03	0.48	0.061	0.12	0.25	0.369	0.04
PP Left	0.45	0.081	0.13	0.42	0.107	0.10	**0.57**	**0.021**	**0.17**	0.48	0.068	0.16
MD												
CB Right	−0.35	0.189	0.07	−0.29	0.275	0.05	−0.43	0.100	0.09	−0.24	0.394	0.04
CB Left	−0.45	0.080	0.13	−0.44	0.086	0.12	−0.41	0.116	0.09	−0.38	0.158	0.10
PP Right	−0.46	0.071	0.13	−0.36	0.168	0.08	−0.49	0.055	0.12	−0.36	0.191	0.09
PP Left	−0.41	0.118	0.10	−0.48	0.061	0.14	−0.31	0.250	0.05	−0.30	0.283	0.06

*Bold values indicate statistical significance (p < 0.05) without correction for multiple comparisons. Squared semipartial correlations are interpreted as small, medium, and large effect sizes when $r_{\text{sp}}^{2}$ ≥ 0.01, 0.09, and 0.25, respectively*.

*CB, cingulum bundle; PP, perforant pathway; FA, fractional anisotropy; MD, mean diffusivity*.

Supplementary analysis of associations between CWIT performance and measures of directional diffusivity within the TBI group (Supplementary Table 3) revealed a significant correlation, with a moderate effect size, between increased RD in the left PP and poorer performance on the WR condition.

As for associations with performance on the CVLT within the TBI group (Table 5), an increased number of streamline points derived from the left PP was associated with better performance on Trials 1–5, SDFR, and LDFR, accounting for a large proportion of variance in performance on these measures. Similarly, DTI-derived FA of the left PP was increased in relation to better performance on Trials 1–5, SDFR, and LDFR of the CVLT, and decreased MD of the right and left PP was associated with better SDFR and LDFR performance; FA and MD accounted for a large proportion of variance in CVLT performance in each of these cases. No relation was found between MD of the left PP and performance on Trials 1–5 of the CVLT; however, decreased MD of the right PP was associated with better performance on this measure and accounted for a moderate proportion of its variance.

**Table 5:** Partial correlations between performance on the California Verbal Learning Test (CVLT) and diffusion parameters.

	**Trials 1–5**			**Short-delay free recall**			**Long-delay free recall**		
	***r*_p_**	***p***	$r_{sp}^{2}$	***r*_p_**	***p***	$r_{sp}^{2}$	***r*_p_**	***p***	$r_{sp}^{2}$
Streamline points									
CB Right	−0.17	0.524	0.02	−0.02	0.937	0.00	−0.17	0.533	0.03
CB Left	0.01	0.958	0.00	0.19	0.474	0.04	0.04	0.897	0.00
PP Right	0.23	0.390	0.04	0.31	0.239	0.09	0.16	0.551	0.02
PP Left	**0.72**	**0.002**	**0.40**	**0.67**	**0.004**	**0.44**	**0.66**	**0.005**	**0.41**
FA									
CB Right	0.27	0.313	0.06	0.36	0.169	0.13	0.40	0.127	0.15
CB Left	0.33	0.216	0.08	0.46	0.077	0.20	**0.51**	**0.043**	**0.25**
PP Right	0.30	0.254	0.07	0.41	0.112	0.16	0.40	0.121	0.15
PP Left	**0.67**	**0.005**	**0.34**	**0.70**	**0.003**	**0.47**	**0.77**	**0.001**	**0.55**
MD									
CB Right	−0.44	0.090	0.15	**−0.58**	**0.019**	**0.32**	**−0.64**	**0.007**	**0.39**
CB Left	−0.50	0.050	0.19	**−0.50**	**0.046**	**0.25**	**−0.69**	**0.003**	**0.44**
PP Right	**−0.50**	**0.048**	**0.19**	**−0.67**	**0.004**	**0.44**	**−0.67**	**0.004**	**0.43**
PP Left	−0.48	0.061	0.18	**−0.52**	**0.039**	**0.26**	**−0.66**	**0.006**	**0.41**

*Bold values indicate statistical significance (p < 0.05) without correction for multiple comparisons. Squared semipartial correlations are interpreted as small, medium, and large effect sizes when $r_{\text{sp}}^{2}$ ≥ 0.01, 0.09, and 0.25, respectively*.

*CB, cingulum bundle; PP, perforant pathway; FA, fractional anisotropy; MD, mean diffusivity*.

No associations between CVLT performance and the number of streamline points of the CB were found (Table 5); however, increased FA of the left CB was related to better LDFR performance. Additionally, decreased MD of the bilateral CB was associated with better performance on CVLT SDFR and LDFR.

**Figure 3:**
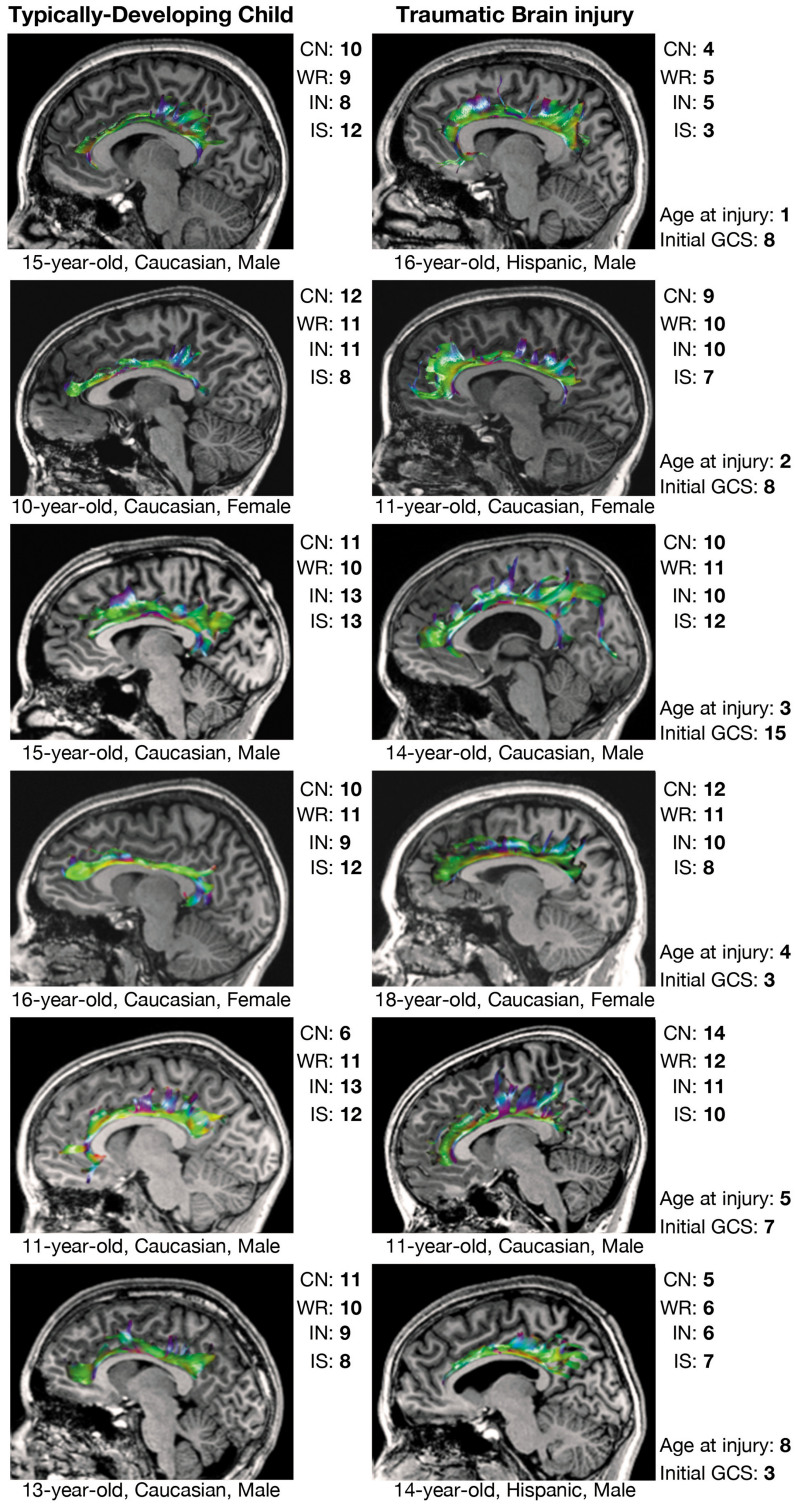
Executive functioning and CB tractography in typically-developing children and children with traumatic brain injury. Executive functioning is reflected by scaled scores (mean = 10 ± 3) on the color naming (CN), word reading (WR), inhibition (IN), and interference/switching (IS) trials of the Delis-Kaplan Executive Function System (D-KEFS) Color-Word Interference Test (CWIT). CB, cingulum bundle; GCS, Glasgow Coma Scale score.

**Figure 4:**
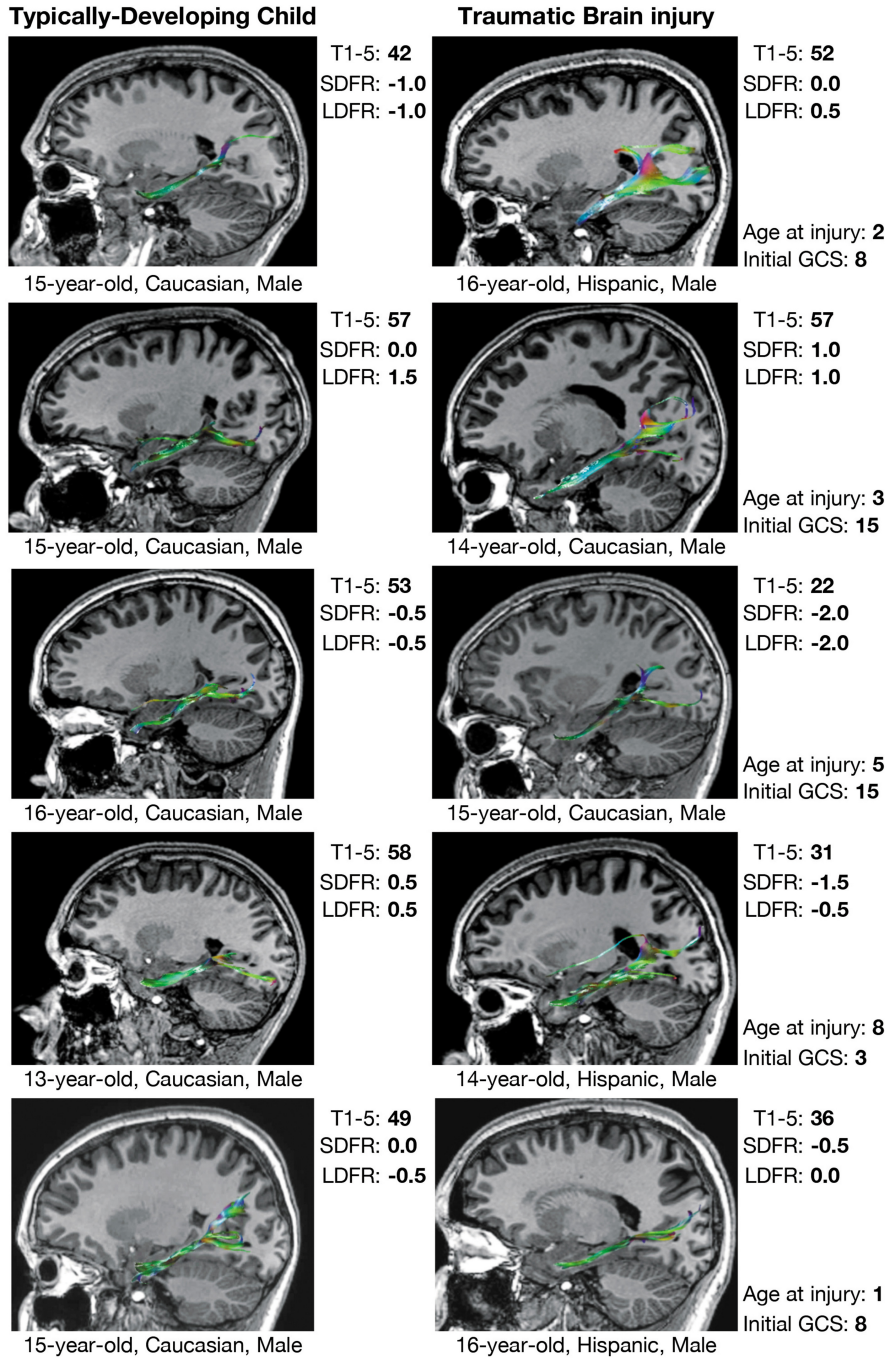
Verbal memory and PP tractography in typically-developing children and children with traumatic brain injury. Verbal memory performance is measured by T-scores (mean = 50 ± 10) on Trials 1–5 (T1–5) and *z*-scores (mean = 0.0 ± 1.0) on short-delay free recall (SDFR) and long-delay free recall (LDFR) trials of the California Verbal Learning Test (CVLT). PP, perforant pathway; GCS, Glasgow Coma Scale score.

Comparisons in CWIT and CVLT performance between select children from the TBI group and demographically-matched children from the TDC group are shown along with tractographic renderings of the CB and PP in Figures 3, 4, respectively. Additionally, graphical representations of the significant correlations reported between DTI metrics and performance on the CWIT and CVLT can be found in the Supplementary Figures.

Supplementary analyses of associations between measures of directional diffusivity and CVLT performance (Supplementary Table 4) revealed significant associations between increased AD of the right PP and poorer SDFR and LDFR performance within the TBI group. Significant associations were also present between increased RD of the left PP and poorer performance on Trials 1–5 of the CVLT, as well as between increased RD of both the PP and CB bilaterally and poorer performance on CVLT SDFR and LDFR. In each of these relationships, fluctuations in directional diffusivity accounted for a large proportion of the variance in CVLT performance.

**Figure 5:**
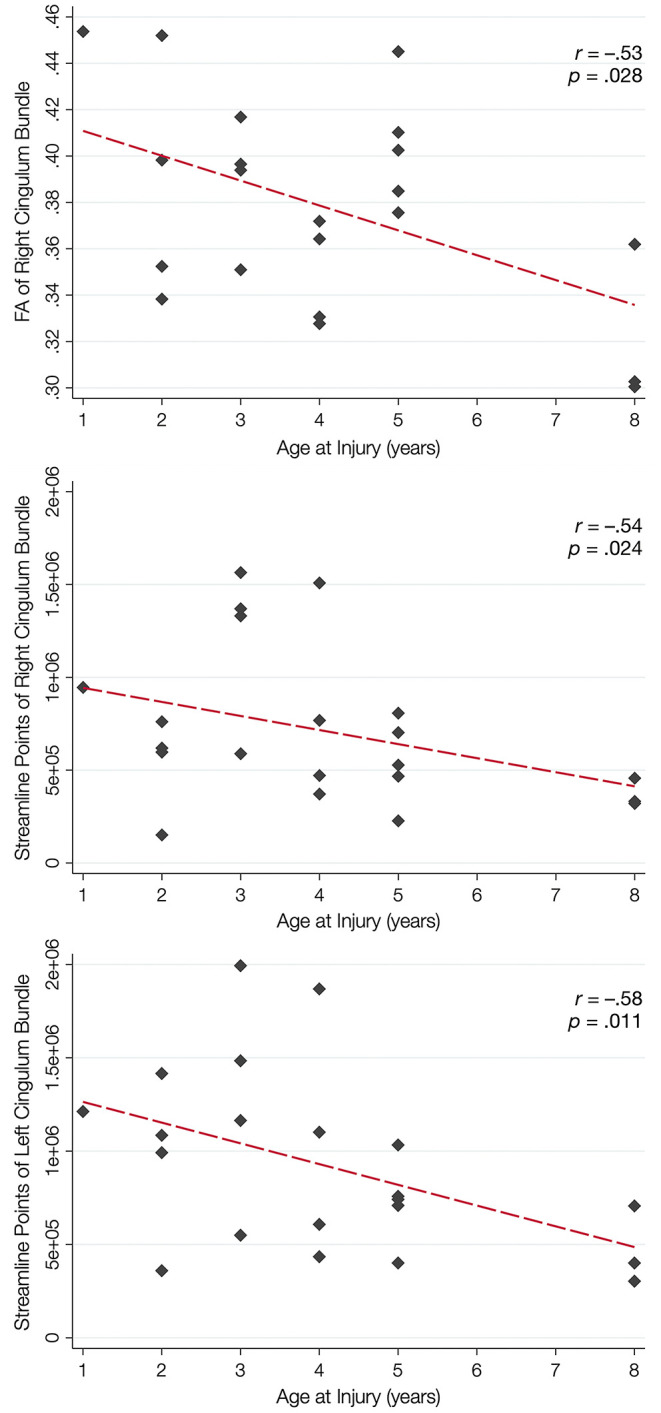
Relation between white matter integrity of the CB and age at brain injury. In the top panel, younger age at injury is associated with increased fractional anisotropy (FA) of the cingulum bundle (CB), but not the perforant pathway (PP; not shown). Younger age at injury is also associated with an increased number of streamline points for the right CB (middle panel) and the left CB (bottom panel) but not the PP (not shown).

Over half of the children in the TBI group exhibited hypertrophic appearance in the anterior region of the CB (Figure 3). Greater hypertrophy was reflected by an increased number of streamline points in the bilateral CB, and this was significantly associated with younger age at injury, accounting for a large proportion of variance (Table 6). Increased FA in the right CB was also associated with younger age at injury (Figure 5); however, MD of the CB was not associated with age at injury. Age at injury was not related to the number of streamline points, FA, or MD of the PP.

**Table 6:** Partial correlations between diffusion metrics and age at injury.

	***r*_p_**	***p***	$r_{sp}^{2}$
Streamline points			
CB Right	**−0.56**	**0.024**	**0.29**
CB Left	**−0.62**	**0.011**	**0.34**
PP Right	−0.38	0.149	0.12
PP Left	−0.03	0.925	0.00
FA			
CB Right	**−0.55**	**0.027**	**0.28**
CB Left	−0.49	0.056	0.20
PP Right	−0.20	0.456	0.04
PP Left	−0.06	0.833	0.00
MD			
CB Right	0.13	0.631	0.02
CB Left	−0.08	0.778	0.01
PP Right	0.13	0.638	0.01
PP Left	0.03	0.909	0.00

*Bold values indicate statistical significance (p < 0.05) without correction for multiple comparisons. Squared semipartial correlations are interpreted as small, medium, and large effect sizes when $r_{\text{sp}}^{2}$ ≥ 0.01, 0.09, and 0.25, respectively*.

*CB, cingulum bundle; PP, perforant pathway; FA, fractional anisotropy; MD, mean diffusivity*.

Supplementary analyses further revealed that younger age at injury was significantly associated with higher AD in the left CB (Supplementary Table 5), and a moderate effect size was present for the right CB. A moderate effect size was also present for the relationship between younger age at injury and increased RD in the right CB. No relationships were present between age at injury and AD or RD of the PP.

## Discussion

We explored the effects of early TBI on later structural development and functionality of the CB and PP. We observed anticipated decreases in FA and increases in MD in both pathways, which had moderate-to-large effect sizes, along with poorer cognitive performance on measures of executive functioning and verbal memory in the participants who experienced a childhood TBI when compared to those in the TDC group. Our results also suggest that in those with early childhood TBI, cognitive performance and age at the time of injury are differentially related to changes in streamline density and tract integrity of the CB versus the PP.

These results are consistent with previous studies reporting impaired cognitive outcome due to injuries sustained to the CB and PP (15, 16, 31). Projections from the CB to the prefrontal cortex (also late developing), posterior cortex, and limbic regions surrounding the corpus callosum promote communication between these regions (37). The function of the CB is linked to cognitive control, working memory, and executive functioning, and these functions are compromised when the CB is damaged (15, 38); hence, our inclusion of the CWIT, which is a sensitive measure of components of executive functioning, including cognitive inhibition and response flexibility (23). The PP has been linked to verbal episodic memory (16), and previous studies have reported that performance on the CVLT, a sensitive measure of verbal learning and memory, is impaired by injuries sustained to the PP (39–42); our results are consistent with these findings.

Prior DTI studies in moderate-to-severe TBI generally report overall decreases in FA and increases in MD, which are reflected by diminished tractographic renderings of WM tracts in the affected areas (15, 16). However, despite these anticipated group differences, careful visual inspection of our data revealed unexpected, qualitative differences in the tractography-derived pathways of children who sustained early injuries. Using quantitative diffusion tractography, which evaluates the number of distinct streamlines that pass through an ROI, we observed elaborated, hypertrophic pathways that were more prominent in children who experienced TBI at a younger age. These elaborations were particularly evident in the CB and, to a lesser extent, the PP; however, the relation of the DTI-derived parameters extracted from these tracts demonstrated a disparate relation to age at injury and performance on cognitive testing. An increased number of tract streamlines in the bilateral CB was strongly associated with younger age at injury, particularly in late-developing frontal regions, though the number of streamlines was not related to executive functioning, as measured by the CWIT (Figure 3). In contrast, the streamline density of the PP was not related to age at injury; rather, a greater number of streamline points, higher FA, and lower MD in the left PP was associated with better verbal memory performance on the CVLT. In the right PP, lower MD, without change in streamline density or FA, was also associated with improved verbal recall ability. The positive relationship observed between the number of PP streamlines and verbal memory may illustrate adaptive neuroplasticity that occurs following damage to an early-developing tract, which was relatively mature at the time of injury. In contrast, the absence of such a relationship in the CB may reflect the long-term, adverse effects of an injury sustained to this late-developing tract and its support of both executive functioning and memory.

There are several potential explanations for changes in WM integrity following pediatric TBI in children with differing ages at the time of injury. These findings may merely represent the heterogeneity associated with TBI, where injury severity influences the extent of axonal injury, with partial injury permitting the myelin reconstitution associated with recovery (10, 43). Furthermore, apparent axon regeneration has been reported in those with late recovery from a minimally conscious state (44). In support of this, our supplementary analysis of measures of directional diffusivity revealed that the higher FA and lower MD associated with verbal memory performance in the TBI group are likely driven by decreased RD, largely without change in AD, which suggests that some extent of remyelination of these pathways may have occurred. Remyelination by surviving oligodendrocytes has been shown to occur after trauma (45, 46), particularly to the developing brain, and it has been suggested that this process may have a neuroprotective effect against axonal damage and neurodegeneration (47).

Another possible mechanism underlying these observations in tractography is that potential dynamic changes in pediatric TBI relate to potential injury-induced proliferation of neural progenitor cells (NPCs). Increased expression of NPC markers has been described in human tissue surrounding focal lesions following injury (48), presumably as a restorative response. Two primary hippocampal pathways have been implicated in the generation, migration, and integration of new neurons into local circuitry (49–51): the subgranular zone (SGZ), which neighbors aspects of the PP, and the subventricular zone (SVZ), which borders aspects of the ventricles, striatum, and CB (52, 53). Interestingly, it is these two regions that also reflect the altered tractography observed in our sample.

We found a striking relation between age at injury and the number of CB streamlines generated through tractography. Specifically, we observed that children injured as toddlers generally demonstrated the most elaborated structures, whereas this relation with streamline density of the CB was less apparent in children who were injured at an older age (Figure 3). Age-dependent increases in post-traumatic neurogenic response to injury have been found to be more robust in the immature brain as opposed to the adult or aged brain in experimental models (54), specifically within the SVZ (55). While this response has generally been presumed to underlie greater functional recovery following TBI (49), it has also been shown to result in altered cell migration patterns, which may contribute to long-term cognitive deficits and the phenomenon of growing into an injury after childhood TBI (55). However, the role that neurogenesis and cell migration have in repair following early TBI and whether these responses are beneficial have yet to be determined. Additionally, this response may also differ regionally; although the generation and migration of neurons remains most apparent in early childhood (49, 55), levels of neural proliferation may remain substantially higher in the SVZ (which borders the CB) as compared to the SGZ (which borders the PP) as the individual matures. This is consistent with our observation that the elaboration of the CB in children with early TBI appeared more age-dependent than the PP. The CB is one of the latest-developing tracts within the human brain (56), which may render it more prone to developmental alteration and myelination following TBI. On the contrary, the PP is an earlier-developing tract that projects from the entorhinal cortex to all fields of the hippocampal formation (57, 58).

### Limitations and Future Directions

Despite the significant findings and moderate-to-large effect sizes, a larger sample is needed to replicate and confirm our observations, especially in view of the heterogeneous pathophysiology in moderate-to-severe TBI in this age range and the long-term effects on neurodevelopment. Although the exclusion of inflicted injury limited recruitment of children who sustained a TBI during infancy, this approach enabled us to focus on children who sustained a single TBI that was well-specified for age at injury without confounding by repetitive head trauma. We also acknowledge the wide range of the time-since-injury interval and age at follow-up in this preliminary study; however, one of the strengths of the study is the close matching of demographic characteristics between the TBI and TDC groups, which mitigated the number of confounding variables that could influence the results of our study. Another limitation of the study is the heterogeneity in mechanism of injury across our sample, as well as the location and severity of focal injury (Table 2). Although we examined structures considered to be important in verbal memory and executive functioning, as well as those in proximity to NPC migration, there may be additional sites of injury that contribute to cognitive deficit and structural change. Longitudinal DTI analysis would further elucidate the integrity of WM and its development throughout the injury-to-imaging time interval and inform the evolution of long-term structural and cognitive outcomes within the TBI group. We also appreciate that individual outcome after early TBI is affected by unique environmental factors, such as stress (2), as well as accessibility and quality of therapeutic intervention, genetic factors, and pre-existing psychological and behavioral disorders that were not assessed in this study. Additionally, single tensor-based methods, such as DTI, are neither able to account for complex architecture or crossing fibers within a voxel nor to determine the accurate origin and destination of fibers (59–61). Furthermore, DTI-derived metrics may be affected by several factors related to image acquisition and preprocessing (60, 62–66). More advanced diffusion-weighted imaging approaches have more recently emerged that demonstrate improvements over the shortcomings of DTI (32), such as high-angular resolution diffusion imaging (67, 68), diffusion spectrum imaging (69), and generalized *q*-sampling imaging (70); thus, future studies should take advantage of these techniques whenever possible. Finally, the present findings are limited by the fact that streamline density is largely dependent on the fiber tracking algorithm used, thus it is not a true reflection of the underlying fiber density (32); however, steps were taken to standardize the fiber tracking algorithm and control for any influence of data quality. For these reasons, we do not believe that the importance of the present findings is undermined. We strongly urge, however, that caution is taken when generalizing the present findings to other samples, particularly when other fiber tracking algorithms are used for analysis.

## Conclusion

The aim of this study was to investigate neuroplasticity and its implications for WM recovery and cognitive outcome after early childhood TBI. As expected, we found evidence that pediatric TBI disrupts WM structure and function, though age at injury may play a significant role in the post-injury development of brain structure. DTI analyses did not fit the conventional tenet of static pathology following TBI, rather these analyses support the possibility of fundamentally altered development in some children who are injured at a very young age. The present findings also challenge the concept that post-trauma indices of brain structure directly relate to functional outcome in an expected pattern.

## Data Availability Statement

The raw data supporting the conclusions of this article will be made available by the authors, without undue reservation.

## Ethics Statement

The studies involving human participants were reviewed and approved by the Baylor College of Medicine Institutional Review Board and the University of Arkansas for Medical Sciences Institutional Review Board. Written informed consent to participate in this study was provided by the participants' legal guardian/next of kin.

## Author Contributions

EW, IH, EB, and SM conceptualized and designed the study. EW, BB, LE-C, MA, and MM assisted with data collection and recruitment procedures. MM also acted as the project and clinical data manager. JH was responsible for reviewing raw imaging data for incidental findings. ZC was responsible for the development and quality assurance of the neuroimaging protocol, and analysis of the neuroimaging data was performed by EW, IH, JF, BB, and LH. MA and LE-C were the site principal investigators. HSL and LN-H were the principal investigators for the entire study. EW, LN-H, and HSL performed project oversite and supervision. EW, IH, HML, JF, JM, SM, JH, LE-C, and MA drafted the manuscript. EB, LN-H, and HSL critically reviewed the manuscript. HML and EW finalized the manuscript. HML performed statistical analyses. HML and IH prepared the figures. All authors contributed to the article and approved the submitted version.

## Funding

The study was funded by the National Institute of Neurological Disorders and Stroke (NINDS) grant R21-NS065937 granted to LN-H and HSL.

## Author Disclaimer

The content of this article is solely the responsibility of the authors and does not necessarily represent the official views of the National Institute of Health.

## Conflict of Interest

The authors declare that the research was conducted in the absence of any commercial or financial relationships that could be construed as a potential conflict of interest.

## Publisher's Note

All claims expressed in this article are solely those of the authors and do not necessarily represent those of their affiliated organizations, or those of the publisher, the editors and the reviewers. Any product that may be evaluated in this article, or claim that may be made by its manufacturer, is not guaranteed or endorsed by the publisher.
