# Supplementary material for: A Preliminary DTI Tractography Study of Developmental Neuroplasticity 5–15 Years After Early Childhood Traumatic Brain Injury
Source: Front Neurol. 2021 Dec 23;12:734055. doi: 10.3389/fneur.2021.734055 (PMC8732947; doi:10.3389/fneur.2021.734055)
Supplement: Supplementary file 1 [file Table_1.DOCX]

**Table S1. Between-site DTI metrics for each group.** Hedges's *g* values are interpreted as small, medium, and large effect sizes when |*g|* ≥ 0.20, 0.50, and 0.80, respectively.

|  | Baylor | | Arkansas | | Hedges' *g* |
| --- | --- | --- | --- | --- | --- |
|  | M | SD | M | SD |  |
| Whole Sample^a^ | | | | | |
| Streamline Points |  |  |  |  |  |
| CB Right | 599.09 | 357.38 | 744.42 | 405.70 | -0.38 |
| CB Left | 805.10 | 430.91 | 961.41 | 481.74 | -0.34 |
| PP Right | 139.18 | 112.79 | 220.30 | 170.96 | -0.57 |
| PP Left | 139.38 | 105.09 | 135.30 | 71.46 | 0.04 |
| FA |  |  |  |  |  |
| CB Right | .3880 | .0448 | .3835 | .0257 | 0.12 |
| CB Left | .4174 | .0432 | .4135 | .0381 | 0.10 |
| PP Right | .3276 | .0306 | .3382 | .0381 | -0.31 |
| PP Left | .3231 | .0330 | .3231 | .0335 | 0.00 |
| MD |  |  |  |  |  |
| CB Right | .7664 | .0331 | .7648 | .0235 | 0.05 |
| CB Left | .7404 | .0295 | .7636 | .0317 | -0.75 |
| PP Right | .8170 | .0570 | .8090 | .0290 | 0.17 |
| PP Left | .7894 | .0499 | .8396 | .0413 | -1.06 |
| Typically-Developing Child Group^b^ | | | | | |
| Streamline Points |  |  |  |  |  |
| CB Right | 637.18 | 350.36 | 567.49 | 345.51 | 0.19 |
| CB Left | 821.25 | 396.28 | 813.45 | 488.60 | 0.02 |
| PP Right | 189.43 | 142.70 | 206.58 | 100.62 | -0.13 |
| PP Left | 154.88 | 93.05 | 118.30 | 78.05 | 0.40 |
| FA |  |  |  |  |  |
| CB Right | .3981 | .0297 | .3918 | .0202 | 0.23 |
| CB Left | .4256 | .0321 | .4244 | .0292 | 0.03 |
| PP Right | .3366 | .0178 | .3487 | .0102 | -0.77 |
| PP Left | .3306 | .0224 | .3268 | .0277 | 0.15 |
| MD |  |  |  |  |  |
| CB Right | .7546 | .0190 | .7613 | .0247 | -0.30 |
| CB Left | .7314 | .0177 | .7502 | .0232 | -0.89 |
| PP Right | .8101 | .0375 | .7995 | .0190 | 0.32 |
| PP Left | .7844 | .0312 | .8298 | .0350 | -1.32 |
| Traumatic Brain Injury Group^c^ | | | | | |
| Streamline Points |  |  |  |  |  |
| CB Right | 564.17 | 375.60 | 901.70 | 406.94 | -0.83 |
| CB Left | 790.29 | 477.60 | 1092.94 | 462.49 | -0.62 |
| PP Right | 93.12 | 45.74 | 232.49 | 221.90 | -0.90 |
| PP Left | 125.18 | 117.29 | 150.41 | 65.85 | -0.24 |
| FA |  |  |  |  |  |
| CB Right | .3787 | .0549 | .3762 | .0288 | 0.05 |
| CB Left | .4100 | .0518 | .4037 | .0440 | 0.12 |
| PP Right | .3193 | .0378 | .3289 | .0510 | -0.21 |
| PP Left | .3162 | .0401 | .3199 | .0393 | -0.09 |
| MD |  |  |  |  |  |
| CB Right | .7772 | .0400 | .7678 | .0235 | 0.26 |
| CB Left | .7486 | .0362 | .7755 | .0347 | -0.73 |
| PP Right | .8234 | .0717 | .8174 | .0346 | 0.10 |
| PP Left | .7939 | .0637 | .8484 | .0465 | -0.92 |

FA = fractional anisotropy; CB = cingulum bundle; PP = perforant pathway; MD = medial diffusivity.

**^a^ Baylor N = 23, Arkansas N = 17**

**^b^ Baylor N = 11, Arkansas N = 8**

**^c^ Baylor N = 12, Arkansas N = 9**

**Table S2. Between-group differences in directional diffusivity metrics.** Bolded values indicate statistical significance (*p* < .05) without correction for multiple comparisons. Negative values of Hedges's *g* indicate lower values in the TBI group relative to the TDC group*.* Hedges's *g* values are interpreted as small, medium, and large effect sizes when |*g|* ≥ 0.20, 0.50, and 0.80, respectively.

|  | TBI (*n* = 21) | | TDC (*n* = 19) | | *t* | *df* ^a^ | *p* | 95% CI | | *g* |
| --- | --- | --- | --- | --- | --- | --- | --- | --- | --- | --- |
|  | *M* | *SD* | *M* | *SD* |  |  |  | *LL* | *UL* |  |
| AD |  |  |  |  |  |  |  |  |  |  |
| CB Right | 1.11 | 0.05 | 1.11 | 0.03 | –0.13 | 35.52 | .899 | –0.03 | 0.02 | –0.04 |
| CB Left | 1.13 | 0.05 | 1.12 | 0.05 | –0.72 | 39.89 | .478 | –0.04 | 0.02 | –0.22 |
| PP Right | 1.12 | 0.06 | 1.12 | 0.04 | 0.06 | 36.14 | .949 | –0.03 | 0.03 | 0.02 |
| PP Left | 1.11 | 0.08 | 1.10 | 0.06 | –0.23 | 39.41 | .818 | –0.05 | 0.04 | –0.07 |
| RD |  |  |  |  |  |  |  |  |  |  |
| CB Right | 0.60 | 0.04 | 0.58 | 0.03 | –1.99 | 34.79 | .054 | –0.05 | 0.00 | –0.60 |
| CB Left | **0.58** | **0.05** | **0.55** | **0.02** | **–2.09** | **30.80** | **.045** | **–0.05** | **0.00** | **–0.63** |
| PP Right | 0.67 | 0.06 | 0.65 | 0.03 | –1.52 | 29.35 | .140 | –0.05 | 0.01 | –0.46 |
| PP Left | 0.67 | 0.06 | 0.65 | 0.03 | –1.16 | 33.29 | .256 | –0.05 | 0.01 | –0.35 |

TBI = traumatic brain injury; TDC = typically-developing child; AD = axial diffusivity; CB = cingulum bundle; PP = perforant pathway; RD = radial diffusivity.

^a^ Welch's approximation was applied to the degrees of freedom.

**Table S3. Partial correlations between performance on the Color-Word Interference Test (CWIT) and directional diffusivity metrics.** Bolded values indicate statistical significance (*p* < .05) without correction for multiple comparisons. Squared semipartial correlations are interpreted as small, medium, and large effect sizes when *r*^2^_sp_ ≥ .01, .09, and .25, respectively.

|  | Color Naming | | |  | Word Reading | | |  | Inhibition | | |  | Inhibition/Switching | | |
| --- | --- | --- | --- | --- | --- | --- | --- | --- | --- | --- | --- | --- | --- | --- | --- |
|  | *r*_p_ | *p* | *r*^2^_sp_ |  | *r*_p_ | *p* | *r*^2^_sp_ |  | *r*_p_ | *p* | *r*^2^_sp_ |  | *r*_p_ | *p* | *r*^2^_sp_ |
| AD |  |  |  |  |  |  |  |  |  |  |  |  |  |  |  |
| CB Right | –.09 | .750 | .00 |  | .05 | .841 | .00 |  | –.24 | .369 | .03 |  | –.33 | .234 | .07 |
| CB Left | –.24 | .373 | .04 |  | –.06 | .816 | .00 |  | –.29 | .284 | .04 |  | –.43 | .111 | .12 |
| PP Right | –.46 | .076 | .13 |  | –.34 | .194 | .07 |  | –.31 | .244 | .05 |  | –.32 | .244 | .07 |
| PP Left | –.28 | .291 | .05 |  | –.36 | .167 | .08 |  | –.08 | .758 | .00 |  | –.13 | .649 | .01 |
| RD |  |  |  |  |  |  |  |  |  |  |  |  |  |  |  |
| CB Right | –.36 | .169 | .08 |  | –.37 | .154 | .08 |  | –.37 | .156 | .07 |  | –.10 | .715 | .01 |
| CB Left | –.42 | .107 | .11 |  | –.49 | .053 | .14 |  | –.35 | .187 | .06 |  | –.25 | .363 | .04 |
| PP Right | –.40 | .123 | .10 |  | –.32 | .227 | .06 |  | –.50 | .050 | .13 |  | –.32 | .240 | .07 |
| PP Left | –.45 | .082 | .12 |  | **–.51** | **.045** | **.15** |  | –.41 | .115 | .09 |  | –.37 | .173 | .09 |

AD = axial diffusivity; CB = cingulum bundle; PP = perforant pathway; RD = radial diffusivity.

**Table S4. Partial correlations between performance on the California Verbal Learning Test (CVLT) and directional diffusivity metrics.** Bolded values indicate statistical significance (*p* < .05) without correction for multiple comparisons. Squared semipartial correlations are interpreted as small, medium, and large effect sizes when *r*^2^_sp_ ≥ .01, .09, and .25, respectively.

|  | Trials 1-5 | | |  | Short-Delay Free Recall | | |  | Long-Delay Free Recall | | |
| --- | --- | --- | --- | --- | --- | --- | --- | --- | --- | --- | --- |
|  | *r*_p_ | *p* | *r*^2^_sp_ |  | *r*_p_ | *p* | *r*^2^_sp_ |  | *r*_p_ | *p* | *r*^2^_sp_ |
| AD |  |  |  |  |  |  |  |  |  |  |  |
| CB Right | –.22 | .405 | .04 |  | –.26 | .330 | .07 |  | –.30 | .265 | .08 |
| CB Left | –.27 | .307 | .06 |  | –.14 | .595 | .02 |  | –.30 | .253 | .09 |
| PP Right | –.42 | .102 | .14 |  | **–.53** | **.035** | **.27** |  | **–.57** | **.022** | **.30** |
| PP Left | –.26 | .340 | .05 |  | –.27 | .311 | .07 |  | –.40 | .121 | .15 |
| RD |  |  |  |  |  |  |  |  |  |  |  |
| CB Right | –.39 | .130 | .12 |  | **–.54** | **.030** | **.28** |  | **–.60** | **.014** | **.34** |
| CB Left | –.46 | .075 | .16 |  | **–.53** | **.036** | **.27** |  | **–.67** | **.005** | **.42** |
| PP Right | –.47 | .069 | .17 |  | **–.64** | **.008** | **.39** |  | **–.63** | **.010** | **.37** |
| PP Left | **–.57** | **.021** | **.25** |  | **–.62** | **.010** | **.38** |  | **–.75** | **.001** | **.53** |

AD = axial diffusivity; CB = cingulum bundle; PP = perforant pathway; RD = radial diffusivity.

**Table S5. Partial correlations between directional diffusivity metrics and age at injury.** Bolded values indicate statistical significance (*p* < .05) without correction for multiple comparisons. Squared semipartial correlations are interpreted as small, medium, and large effect sizes when *r*^2^_sp_ ≥ .01, .09, and .25, respectively.

|  | *r*_p_ | *p* | *r*^2^_sp_ |
| --- | --- | --- | --- |
| AD |  |  |  |
| CB Right | –.42 | .106 | .16 |
| CB Left | **–.58** | **.019** | **.28** |
| PP Right | –.05 | .843 | .00 |
| PP Left | .00 | .997 | .00 |
| RD |  |  |  |
| CB Right | .38 | .144 | .14 |
| CB Left | .18 | .509 | .03 |
| PP Right | .19 | .489 | .03 |
| PP Left | .05 | .865 | .00 |

AD = axial diffusivity; CB = cingulum bundle; PP = perforant pathway; RD = radial diffusivity.
