# Supplementary material for: A Preliminary DTI Tractography Study of Developmental Neuroplasticity 5–15 Years After Early Childhood Traumatic Brain Injury
Source: Front Neurol. 2021 Dec 23;12:734055. doi: 10.3389/fneur.2021.734055 (PMC8732947; doi:10.3389/fneur.2021.734055)
Supplement: Supplementary file 2 [file Presentation_1.PPTX]

## Slide 1
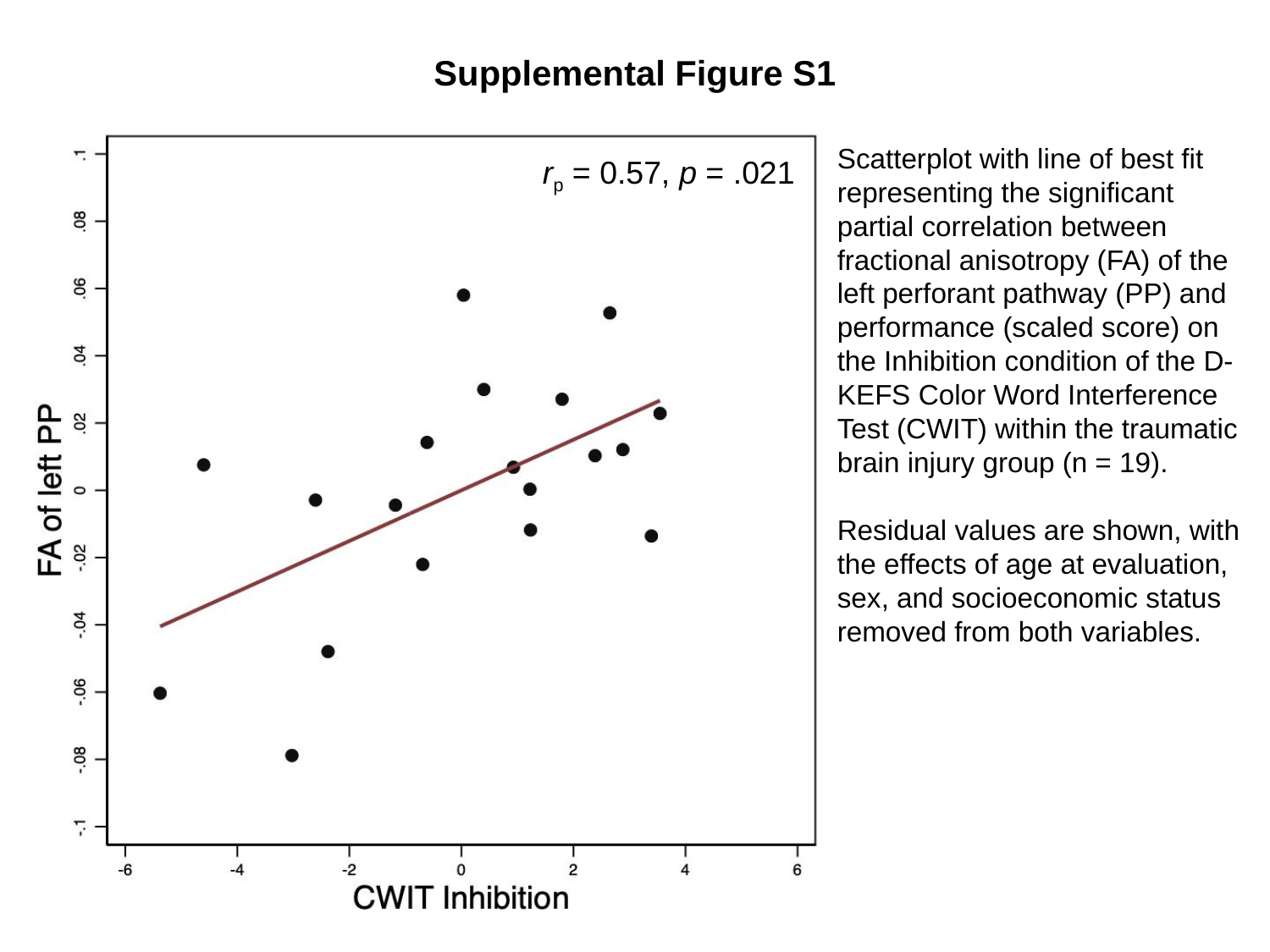

Supplemental Figure S1
Scatterplot with line of best fit representing the significant partial correlation between fractional anisotropy (FA) of the left perforant pathway (PP) and performance (scaled score) on the Inhibition condition of the D-KEFS Color Word Interference Test (CWIT) within the traumatic brain injury group (n = 19).
Residual values are shown, with the effects of age at evaluation, sex, and socioeconomic status removed from both variables.
rp = 0.57, p = .021

## Slide 2
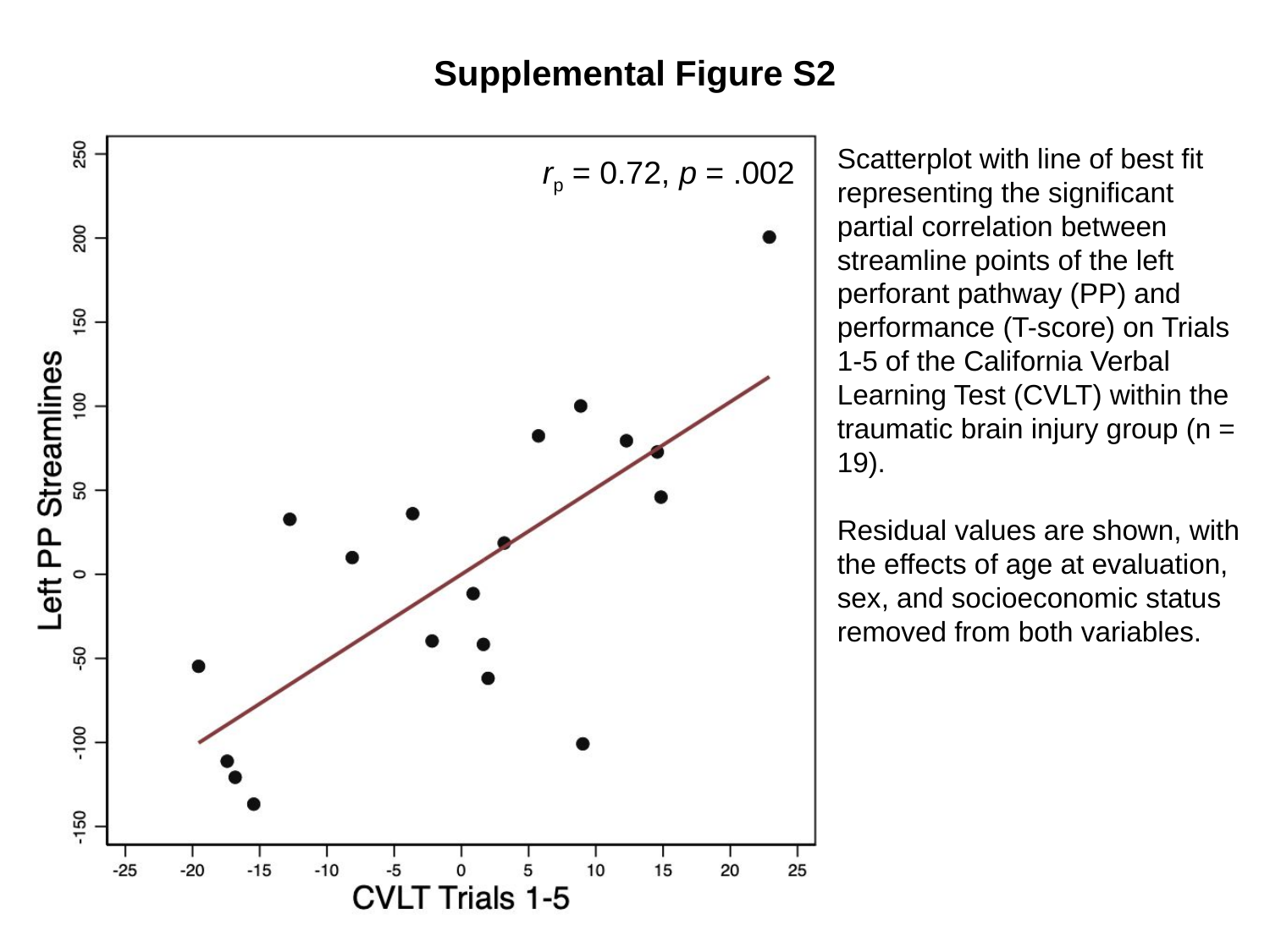

Supplemental Figure S2
Scatterplot with line of best fit representing the significant partial correlation between streamline points of the left perforant pathway (PP) and performance (T-score) on Trials 1-5 of the California Verbal Learning Test (CVLT) within the traumatic brain injury group (n = 19).
Residual values are shown, with the effects of age at evaluation, sex, and socioeconomic status removed from both variables.
rp = 0.72, p = .002

## Slide 3
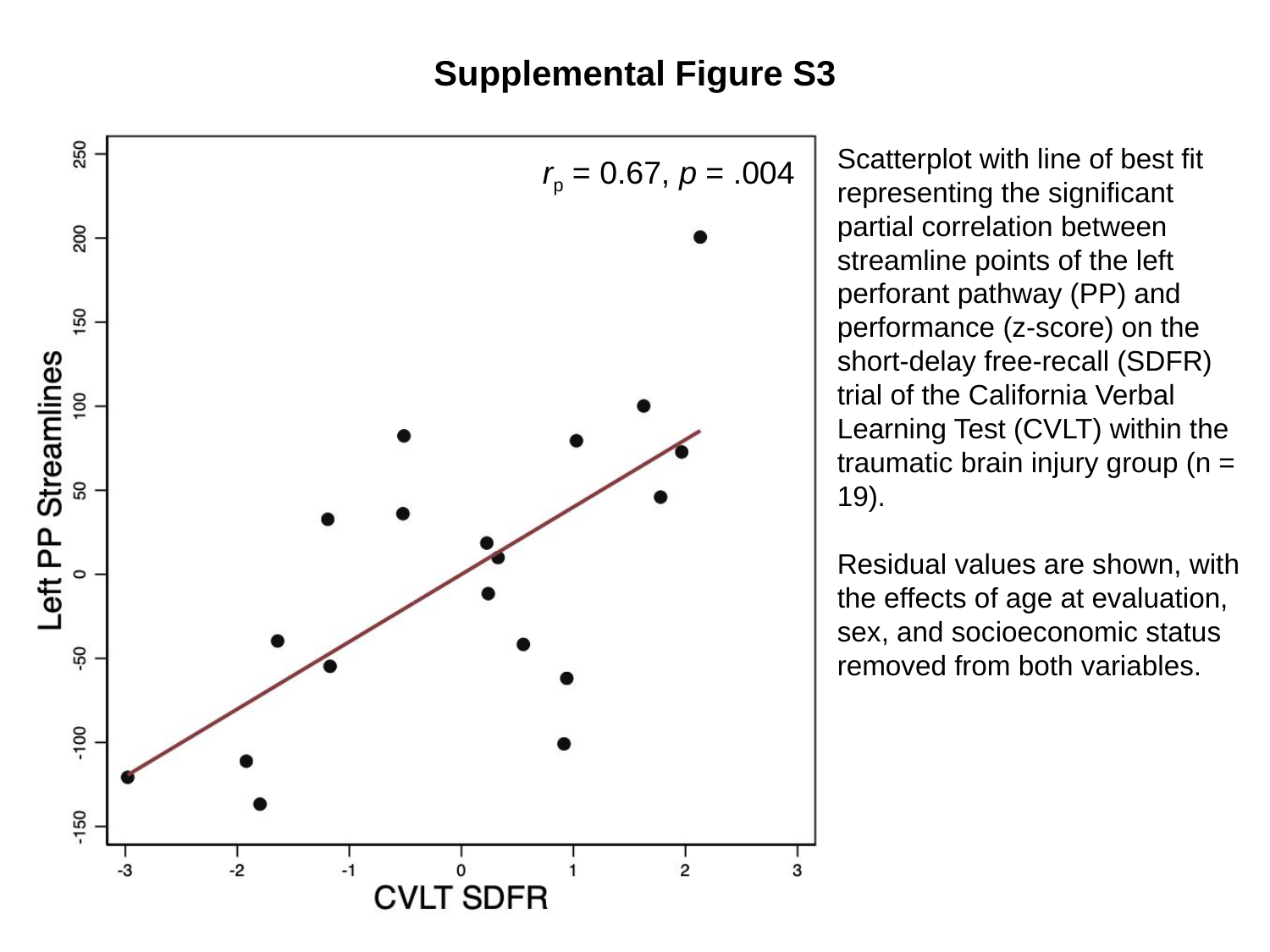

Supplemental Figure S3
Scatterplot with line of best fit representing the significant partial correlation between streamline points of the left perforant pathway (PP) and performance (z-score) on the short-delay free-recall (SDFR) trial of the California Verbal Learning Test (CVLT) within the traumatic brain injury group (n = 19).
Residual values are shown, with the effects of age at evaluation, sex, and socioeconomic status removed from both variables.
rp = 0.67, p = .004

## Slide 4
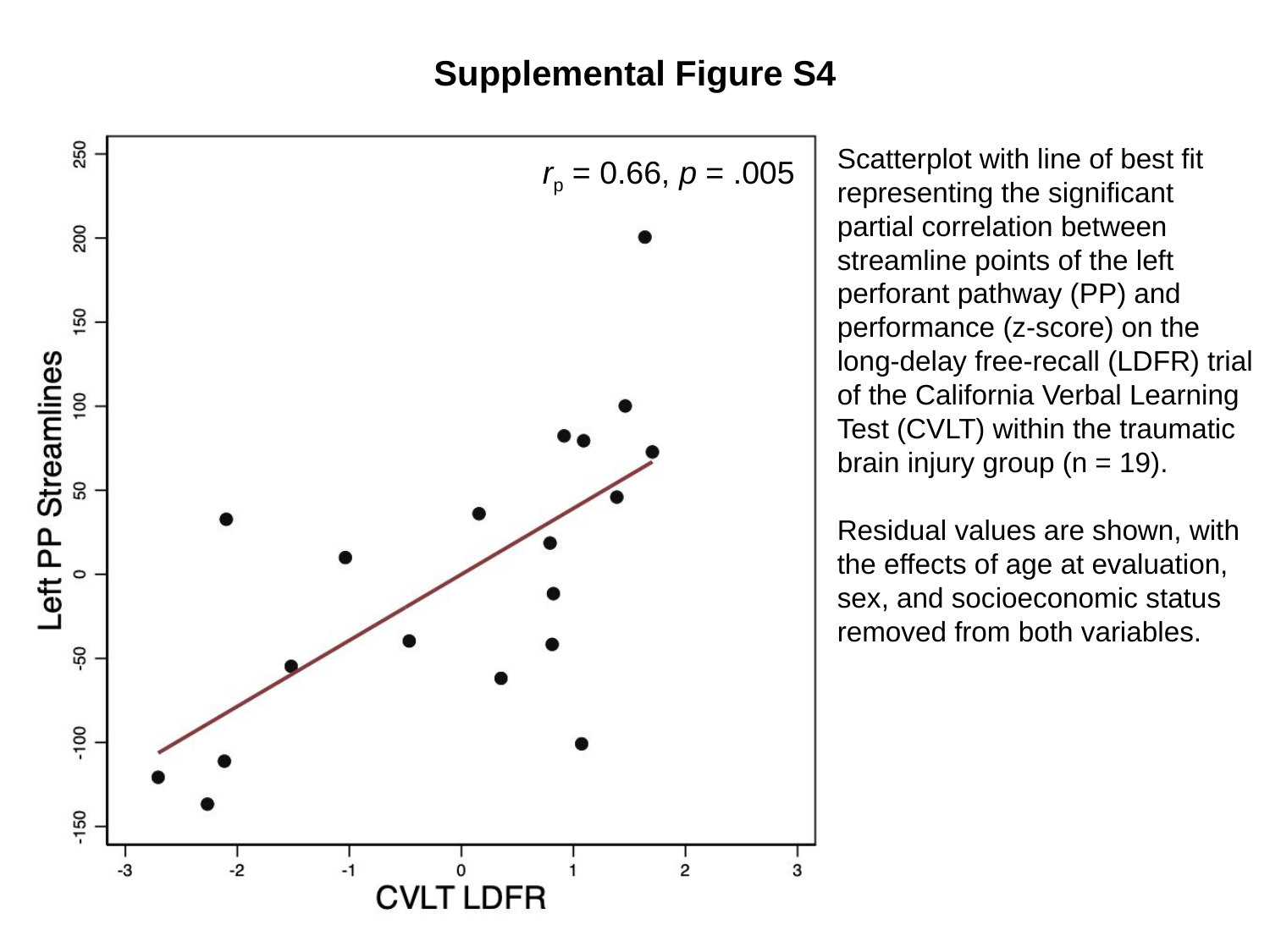

Supplemental Figure S4
Scatterplot with line of best fit representing the significant partial correlation between streamline points of the left perforant pathway (PP) and performance (z-score) on the long-delay free-recall (LDFR) trial of the California Verbal Learning Test (CVLT) within the traumatic brain injury group (n = 19).
Residual values are shown, with the effects of age at evaluation, sex, and socioeconomic status removed from both variables.
rp = 0.66, p = .005

## Slide 5
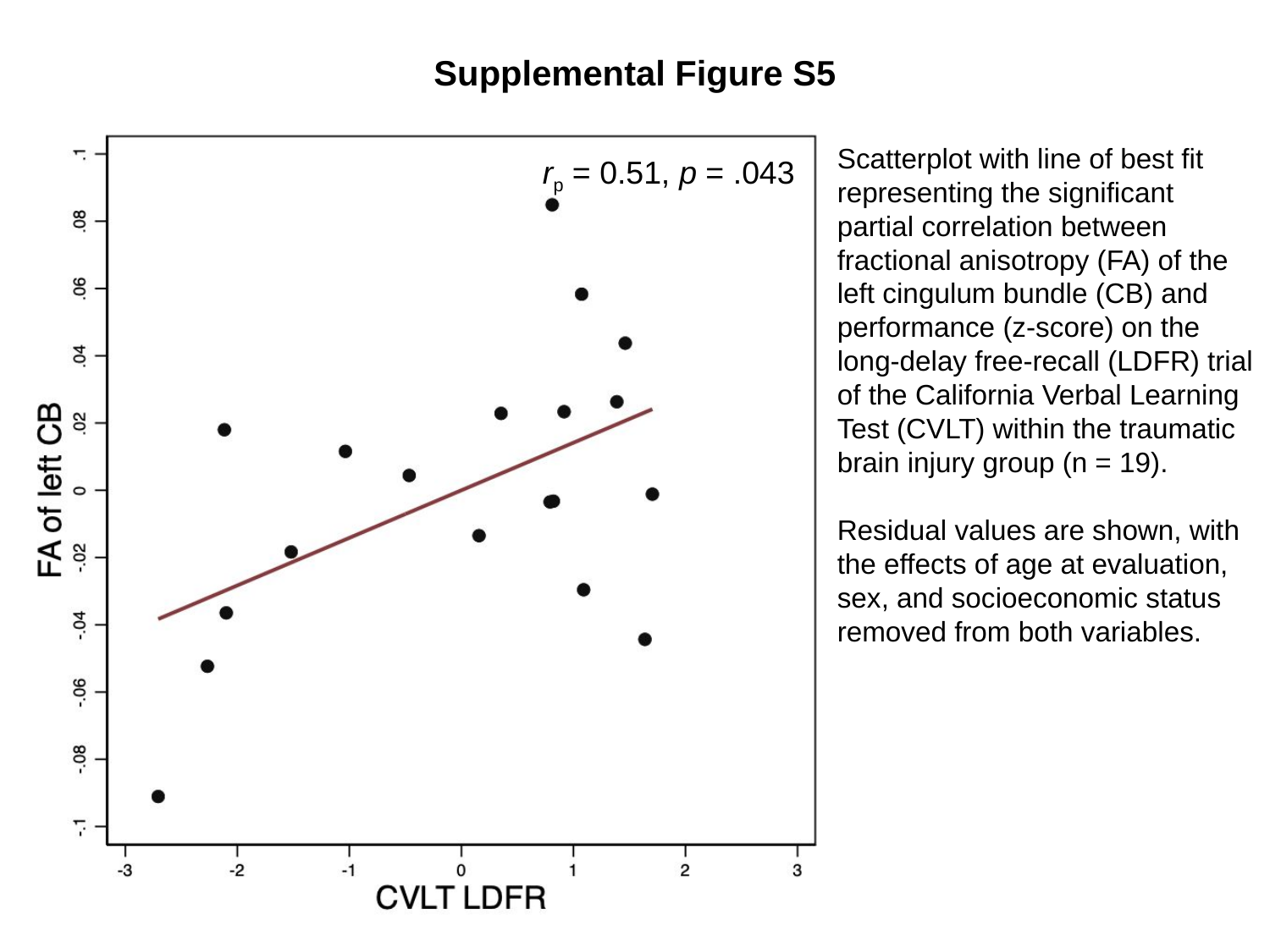

Supplemental Figure S5
Scatterplot with line of best fit representing the significant partial correlation between fractional anisotropy (FA) of the left cingulum bundle (CB) and performance (z-score) on the long-delay free-recall (LDFR) trial of the California Verbal Learning Test (CVLT) within the traumatic brain injury group (n = 19).
Residual values are shown, with the effects of age at evaluation, sex, and socioeconomic status removed from both variables.
rp = 0.51, p = .043

## Slide 6
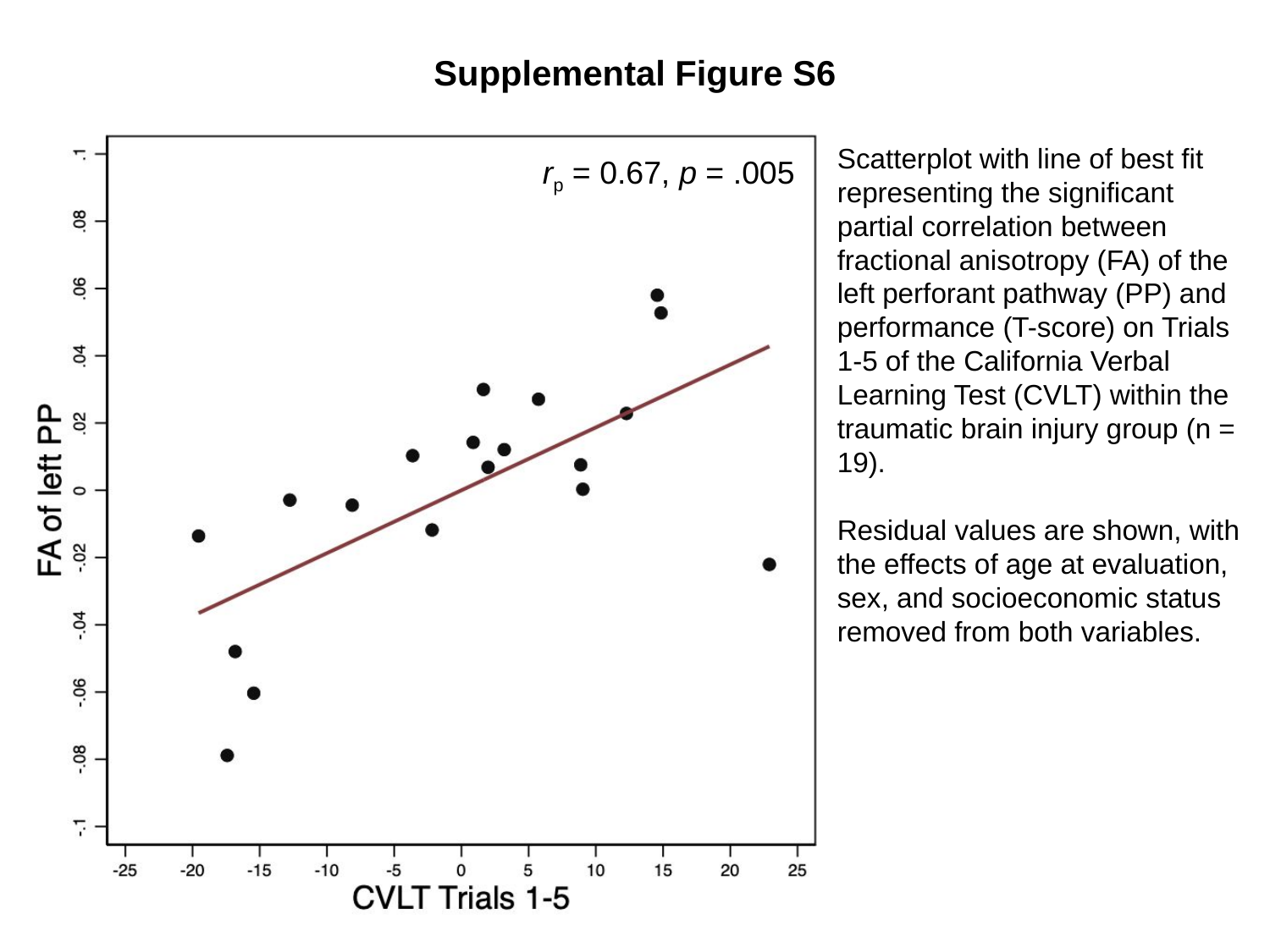

Supplemental Figure S6
Scatterplot with line of best fit representing the significant partial correlation between fractional anisotropy (FA) of the left perforant pathway (PP) and performance (T-score) on Trials 1-5 of the California Verbal Learning Test (CVLT) within the traumatic brain injury group (n = 19).
Residual values are shown, with the effects of age at evaluation, sex, and socioeconomic status removed from both variables.
rp = 0.67, p = .005

## Slide 7
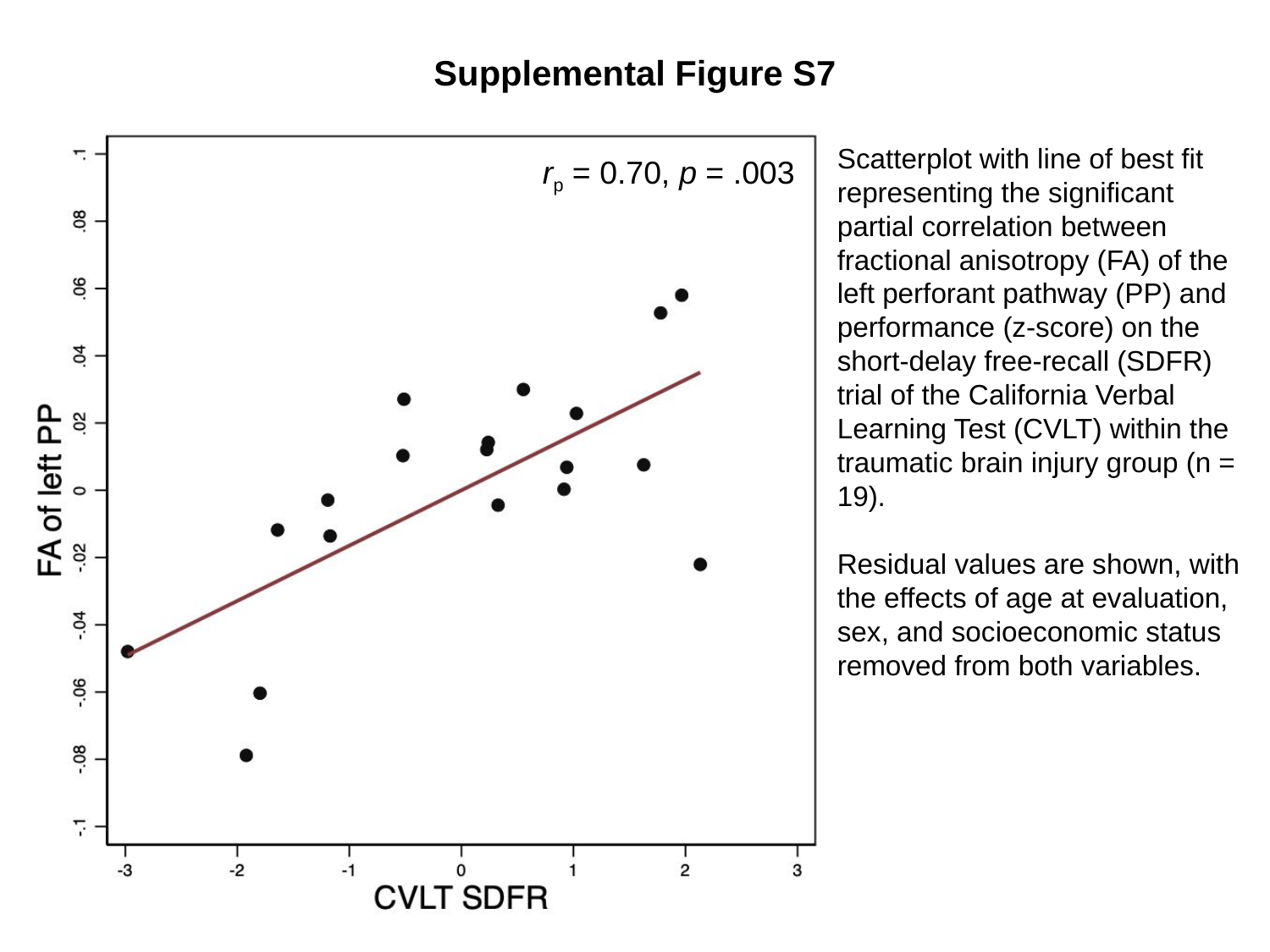

Supplemental Figure S7
Scatterplot with line of best fit representing the significant partial correlation between fractional anisotropy (FA) of the left perforant pathway (PP) and performance (z-score) on the short-delay free-recall (SDFR) trial of the California Verbal Learning Test (CVLT) within the traumatic brain injury group (n = 19).
Residual values are shown, with the effects of age at evaluation, sex, and socioeconomic status removed from both variables.
rp = 0.70, p = .003

## Slide 8
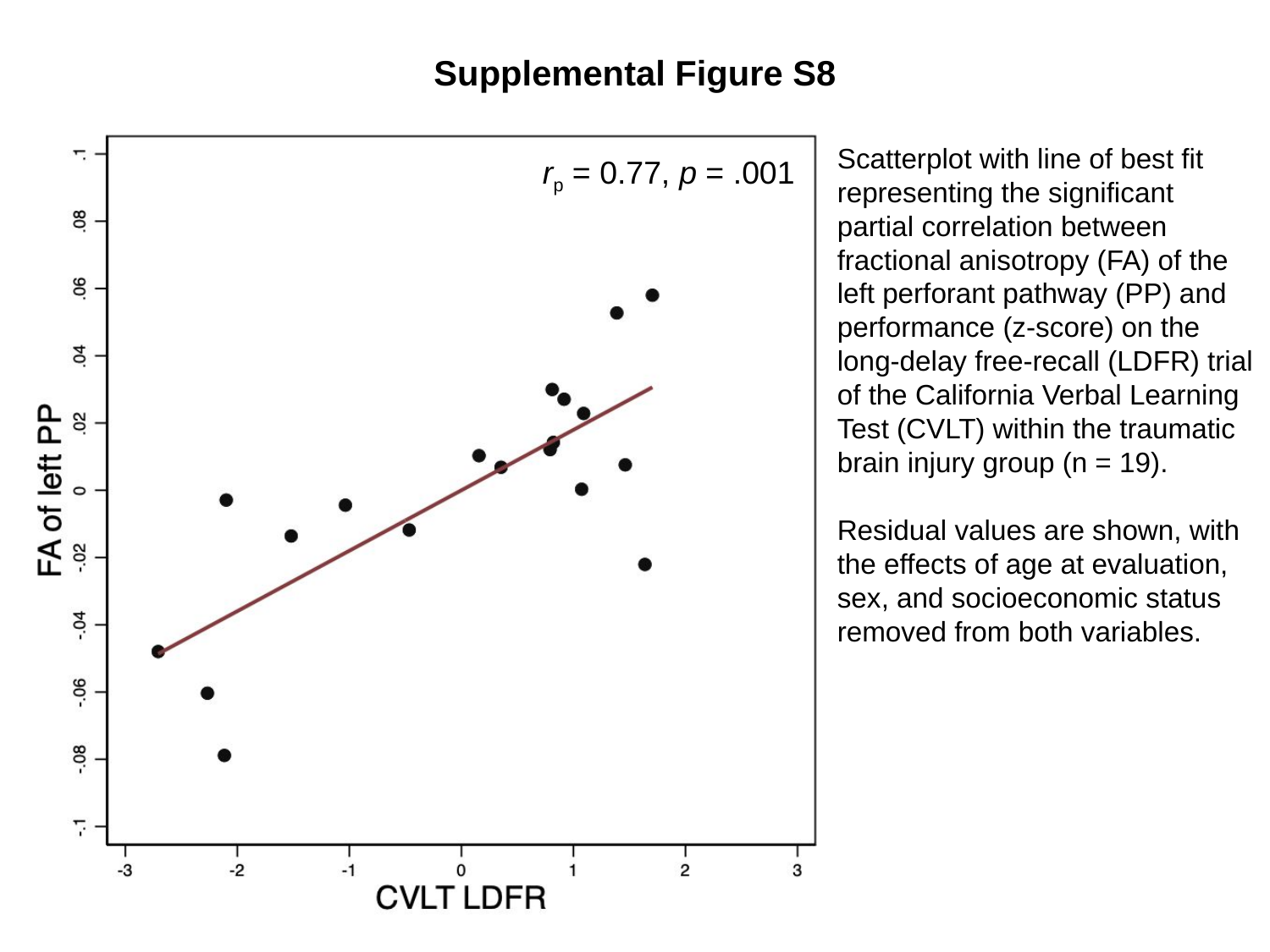

Supplemental Figure S8
Scatterplot with line of best fit representing the significant partial correlation between fractional anisotropy (FA) of the left perforant pathway (PP) and performance (z-score) on the long-delay free-recall (LDFR) trial of the California Verbal Learning Test (CVLT) within the traumatic brain injury group (n = 19).
Residual values are shown, with the effects of age at evaluation, sex, and socioeconomic status removed from both variables.
rp = 0.77, p = .001

## Slide 9
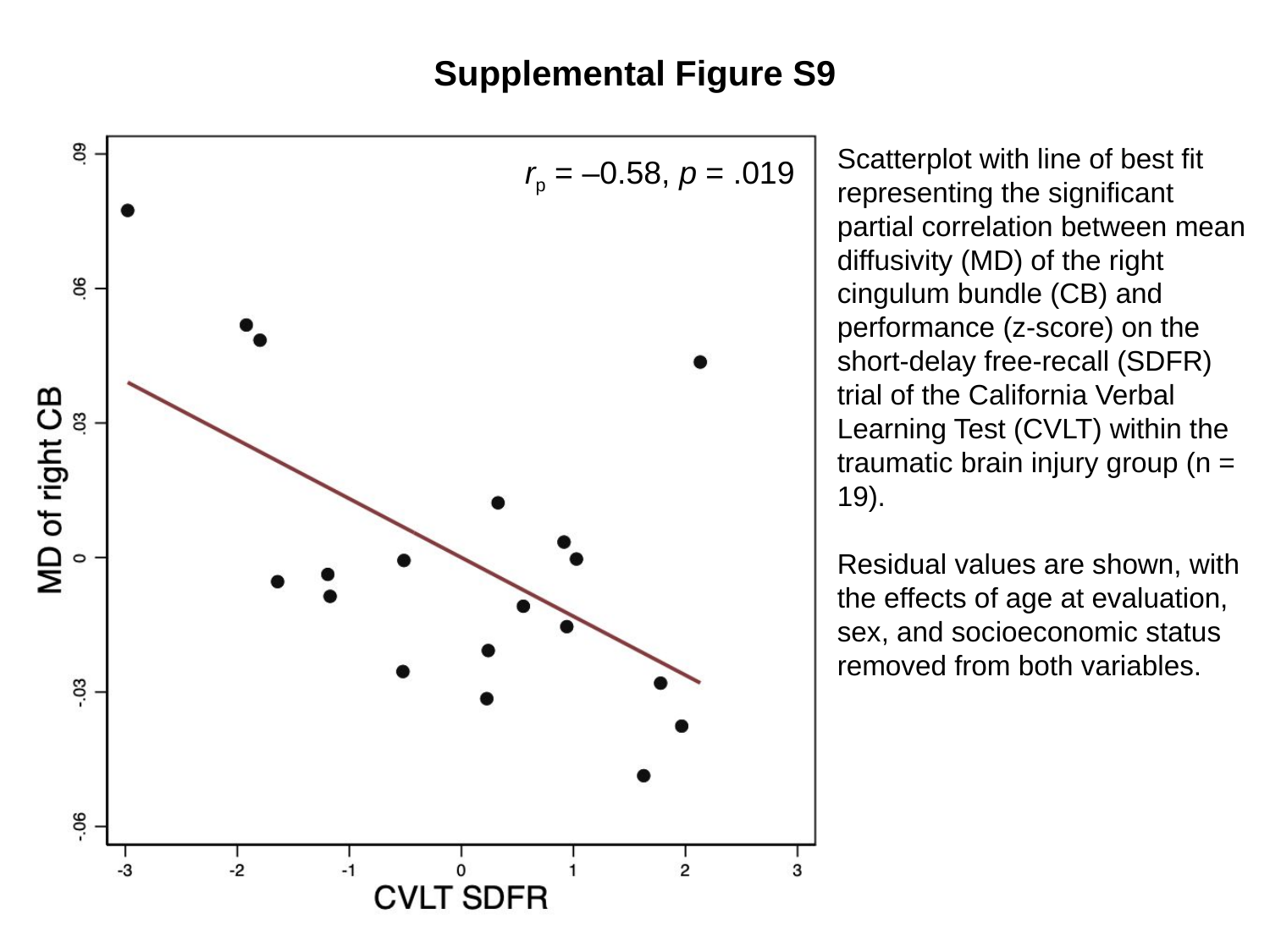

Supplemental Figure S9
Scatterplot with line of best fit representing the significant partial correlation between mean diffusivity (MD) of the right cingulum bundle (CB) and performance (z-score) on the short-delay free-recall (SDFR) trial of the California Verbal Learning Test (CVLT) within the traumatic brain injury group (n = 19).
Residual values are shown, with the effects of age at evaluation, sex, and socioeconomic status removed from both variables.
rp = –0.58, p = .019

## Slide 10
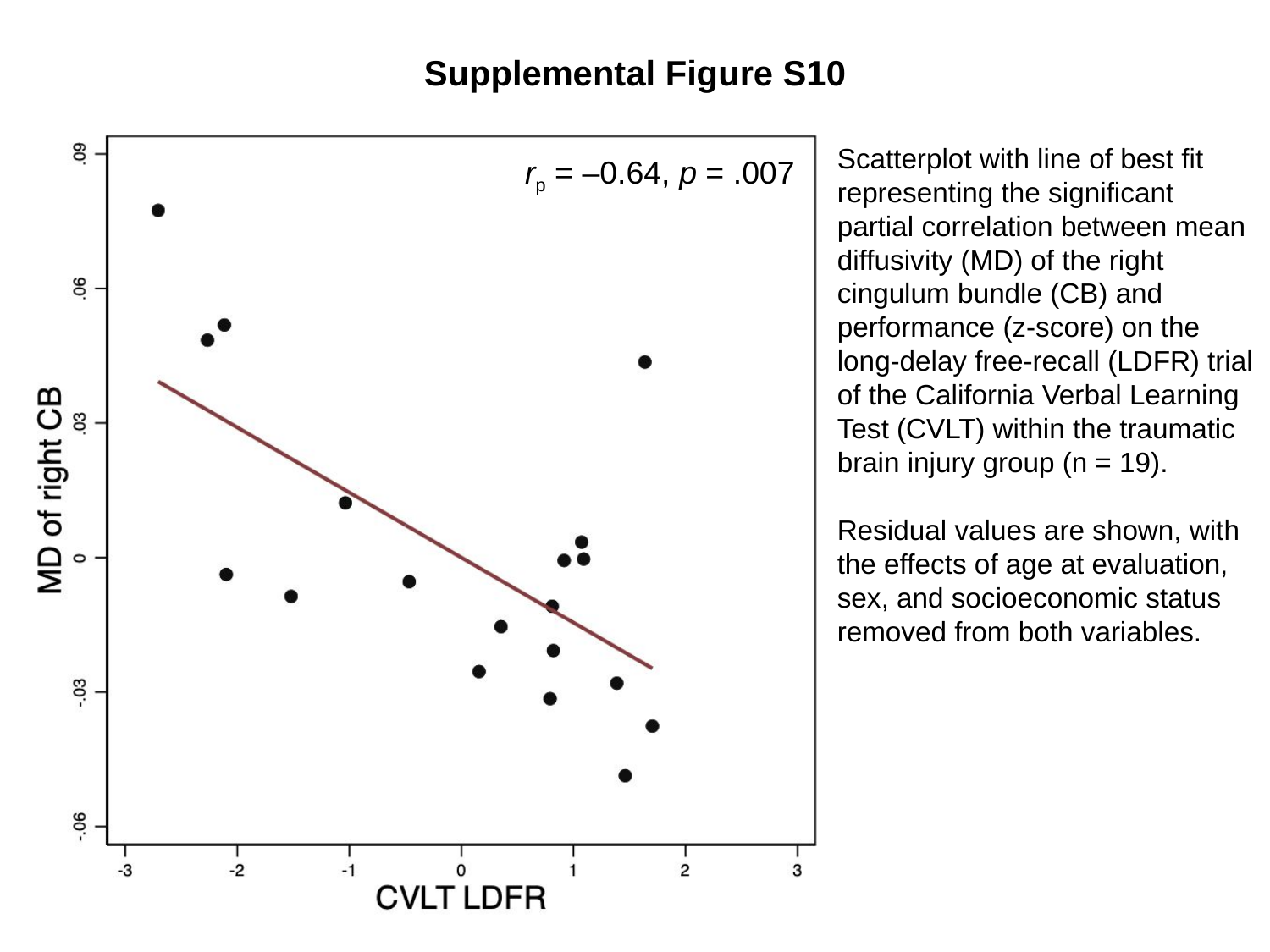

Supplemental Figure S10
Scatterplot with line of best fit representing the significant partial correlation between mean diffusivity (MD) of the right cingulum bundle (CB) and performance (z-score) on the long-delay free-recall (LDFR) trial of the California Verbal Learning Test (CVLT) within the traumatic brain injury group (n = 19).
Residual values are shown, with the effects of age at evaluation, sex, and socioeconomic status removed from both variables.
rp = –0.64, p = .007

## Slide 11
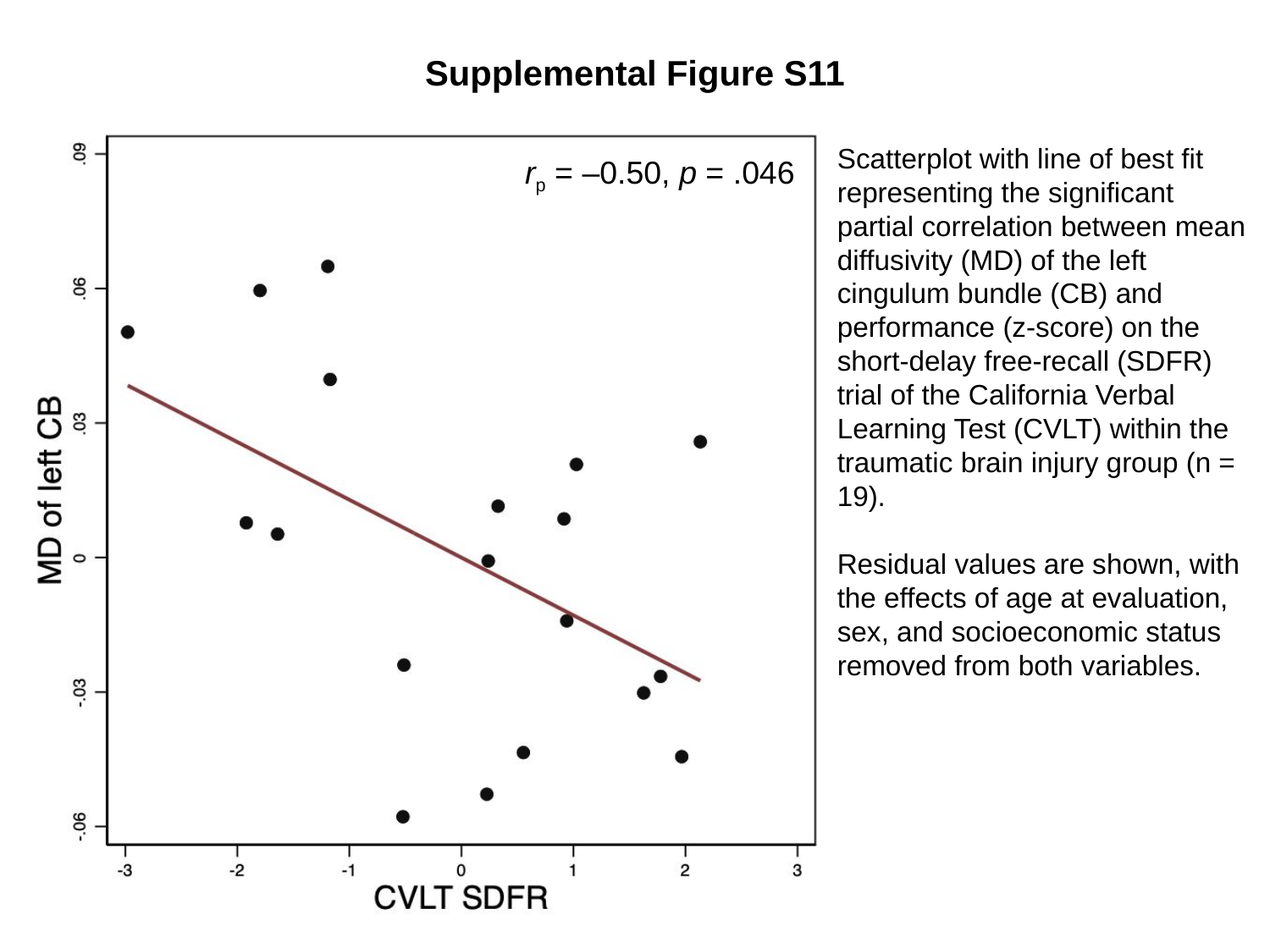

Supplemental Figure S11
Scatterplot with line of best fit representing the significant partial correlation between mean diffusivity (MD) of the left cingulum bundle (CB) and performance (z-score) on the short-delay free-recall (SDFR) trial of the California Verbal Learning Test (CVLT) within the traumatic brain injury group (n = 19).
Residual values are shown, with the effects of age at evaluation, sex, and socioeconomic status removed from both variables.
rp = –0.50, p = .046

## Slide 12
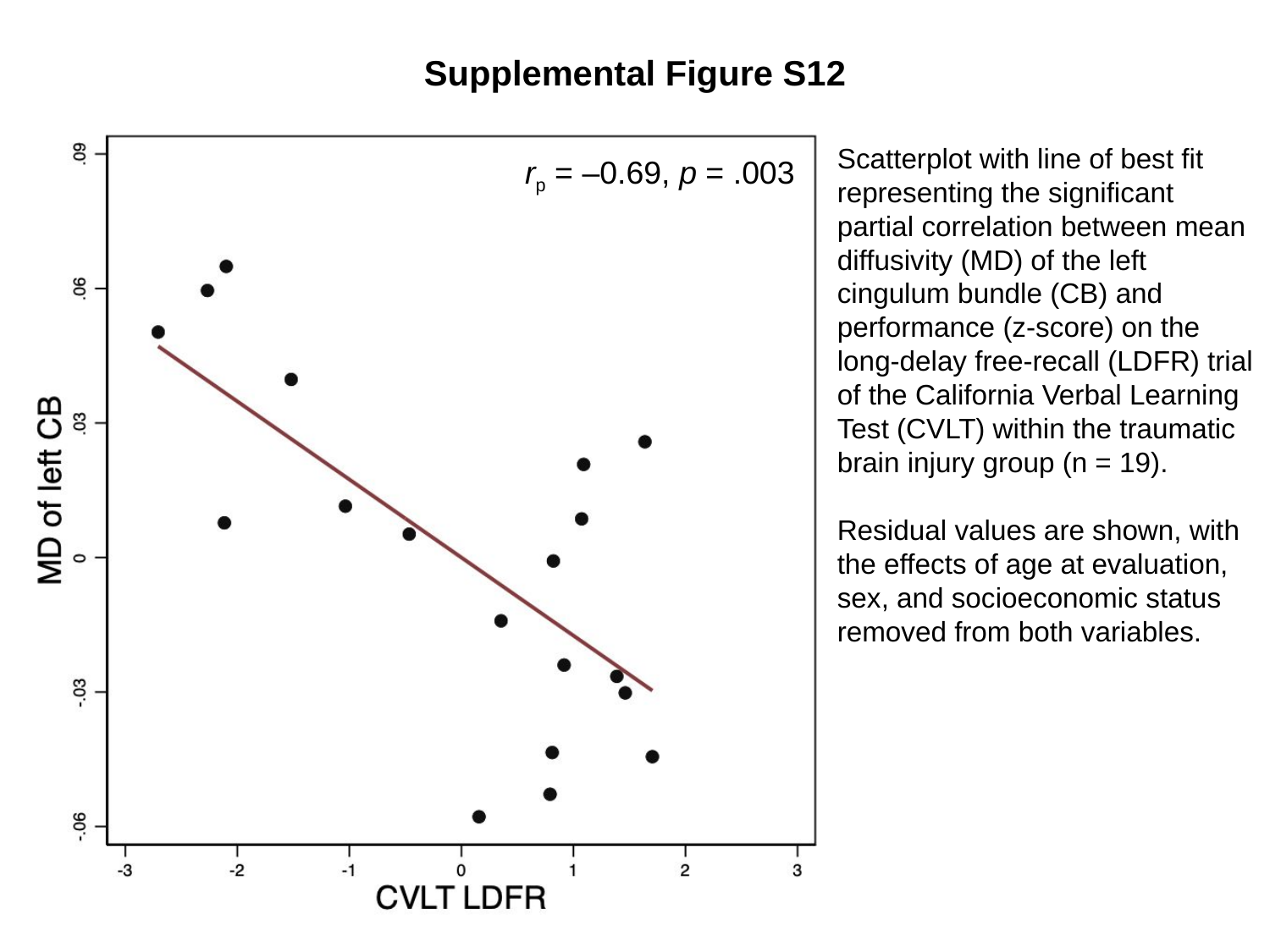

Supplemental Figure S12
Scatterplot with line of best fit representing the significant partial correlation between mean diffusivity (MD) of the left cingulum bundle (CB) and performance (z-score) on the long-delay free-recall (LDFR) trial of the California Verbal Learning Test (CVLT) within the traumatic brain injury group (n = 19).
Residual values are shown, with the effects of age at evaluation, sex, and socioeconomic status removed from both variables.
rp = –0.69, p = .003

## Slide 13
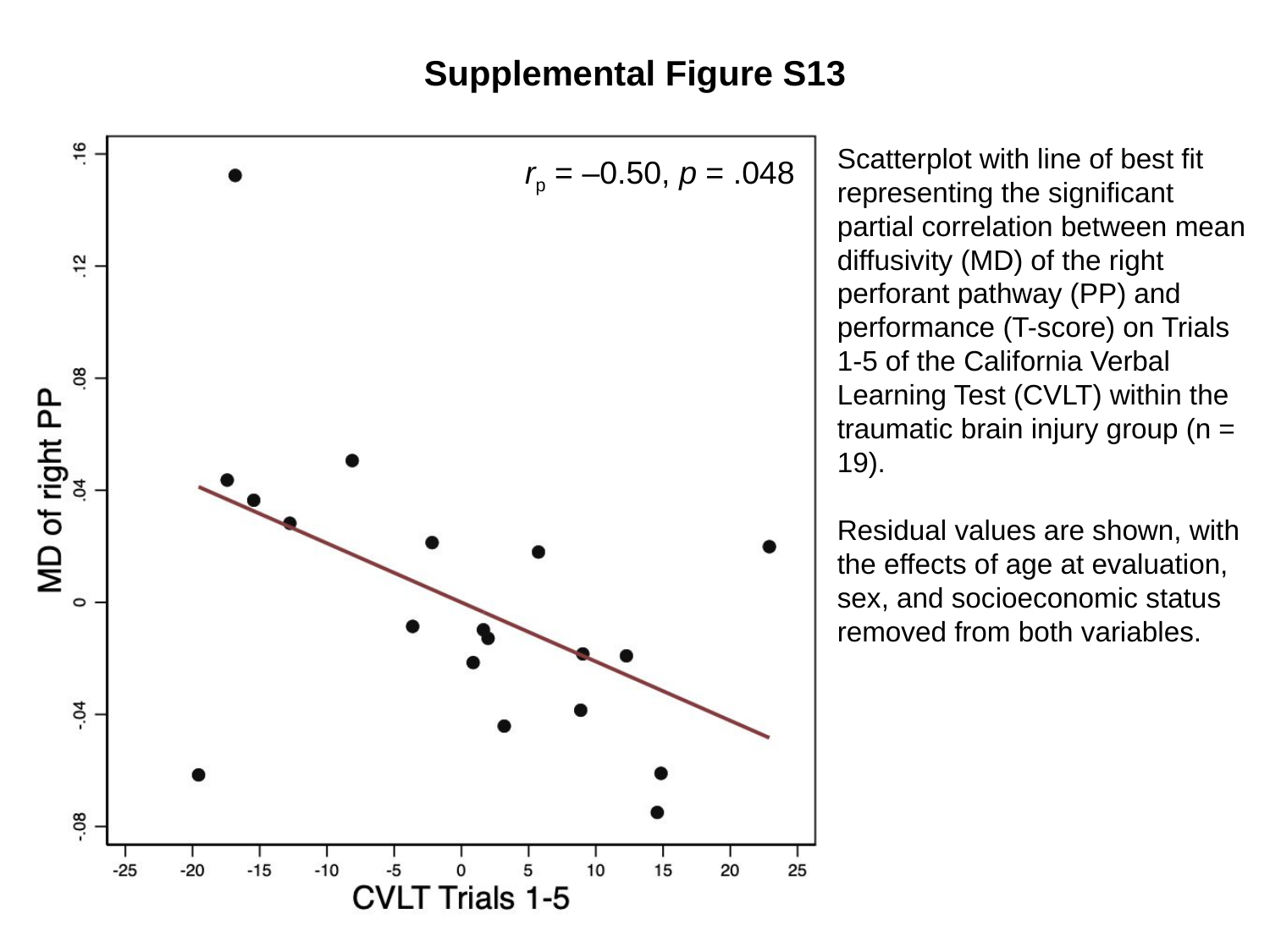

Supplemental Figure S13
Scatterplot with line of best fit representing the significant partial correlation between mean diffusivity (MD) of the right perforant pathway (PP) and performance (T-score) on Trials 1-5 of the California Verbal Learning Test (CVLT) within the traumatic brain injury group (n = 19).
Residual values are shown, with the effects of age at evaluation, sex, and socioeconomic status removed from both variables.
rp = –0.50, p = .048

## Slide 14
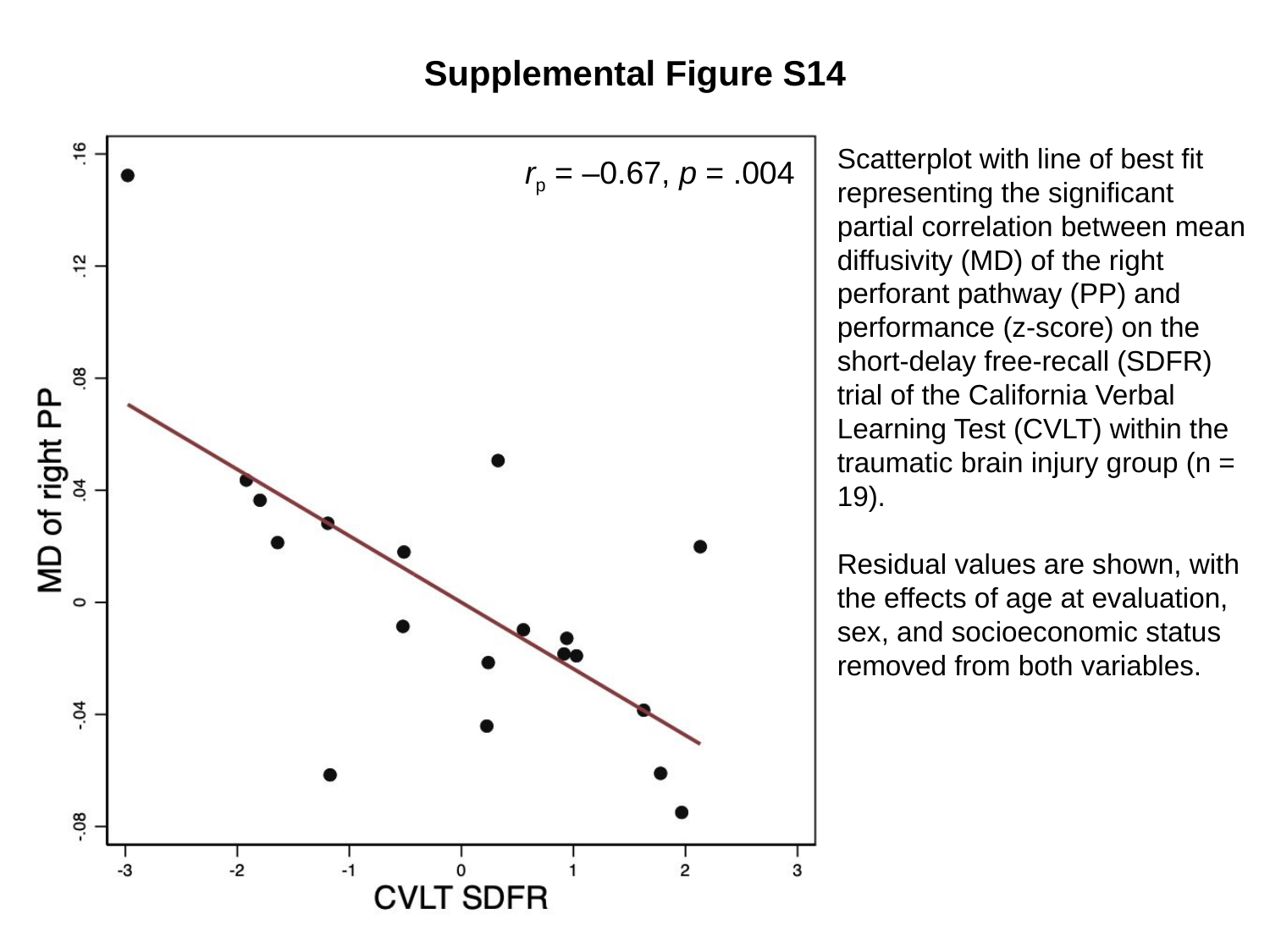

Supplemental Figure S14
Scatterplot with line of best fit representing the significant partial correlation between mean diffusivity (MD) of the right perforant pathway (PP) and performance (z-score) on the short-delay free-recall (SDFR) trial of the California Verbal Learning Test (CVLT) within the traumatic brain injury group (n = 19).
Residual values are shown, with the effects of age at evaluation, sex, and socioeconomic status removed from both variables.
rp = –0.67, p = .004

## Slide 15
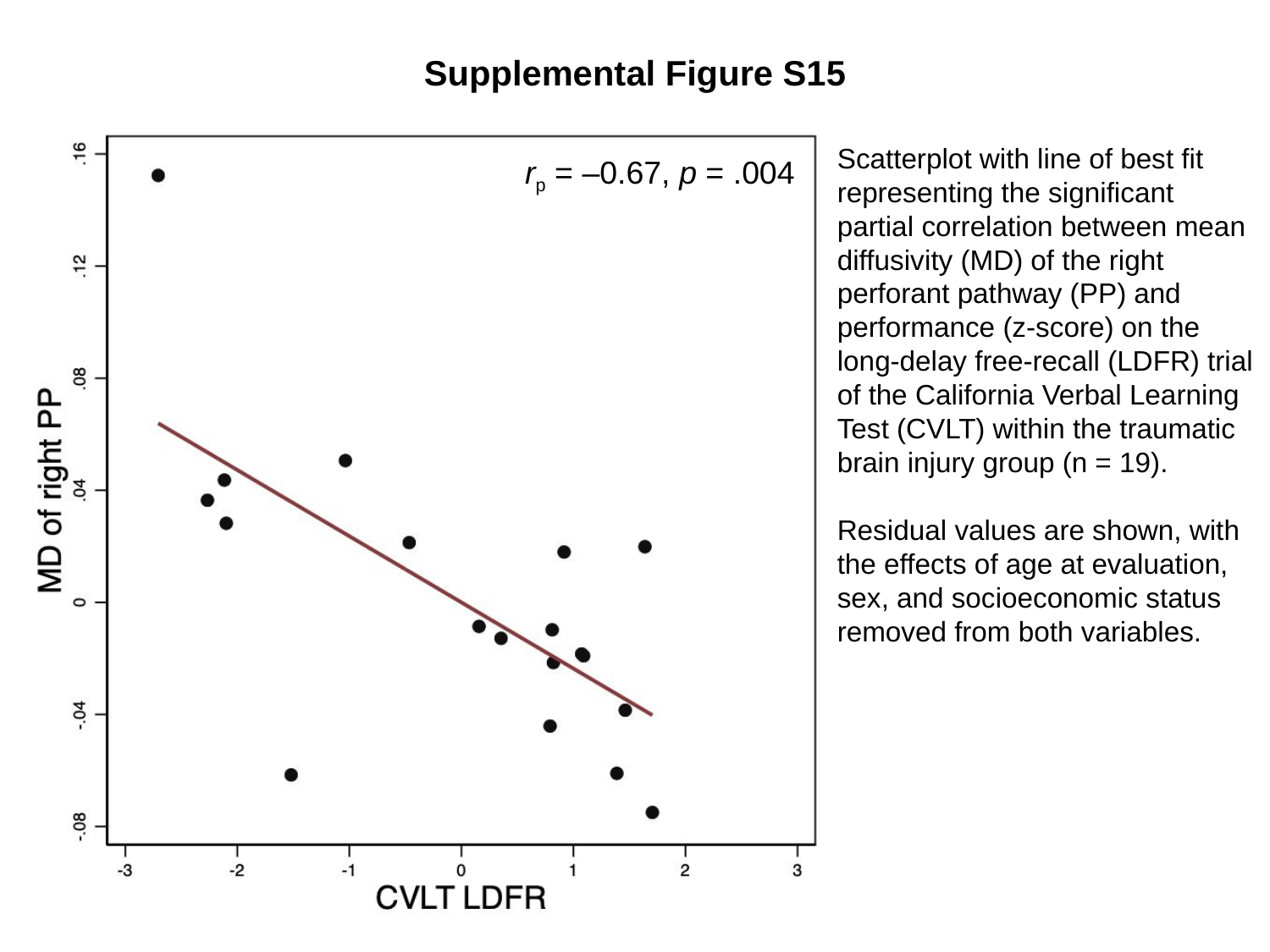

Supplemental Figure S15
Scatterplot with line of best fit representing the significant partial correlation between mean diffusivity (MD) of the right perforant pathway (PP) and performance (z-score) on the long-delay free-recall (LDFR) trial of the California Verbal Learning Test (CVLT) within the traumatic brain injury group (n = 19).
Residual values are shown, with the effects of age at evaluation, sex, and socioeconomic status removed from both variables.
rp = –0.67, p = .004

## Slide 16
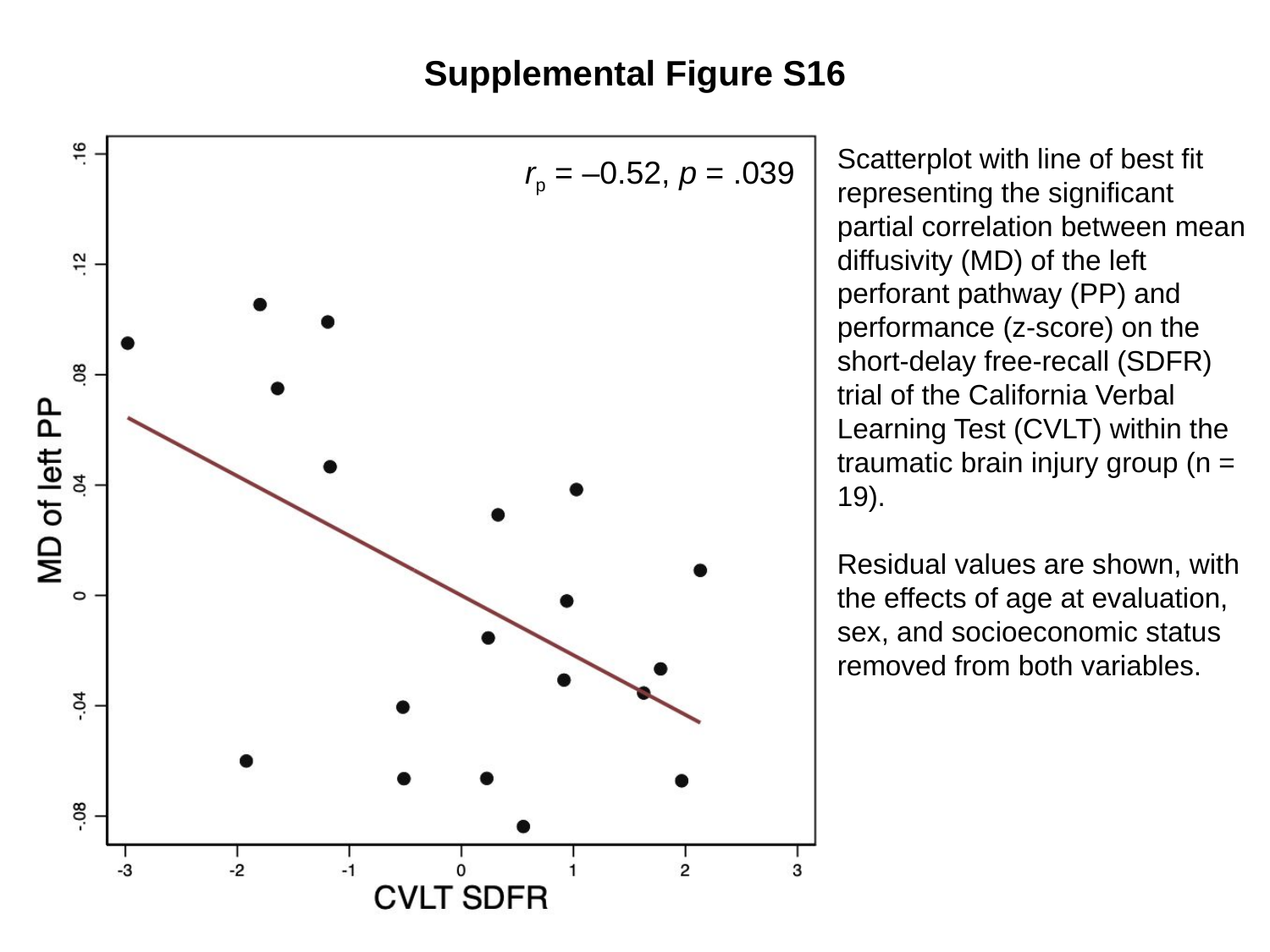

Supplemental Figure S16
Scatterplot with line of best fit representing the significant partial correlation between mean diffusivity (MD) of the left perforant pathway (PP) and performance (z-score) on the short-delay free-recall (SDFR) trial of the California Verbal Learning Test (CVLT) within the traumatic brain injury group (n = 19).
Residual values are shown, with the effects of age at evaluation, sex, and socioeconomic status removed from both variables.
rp = –0.52, p = .039

## Slide 17
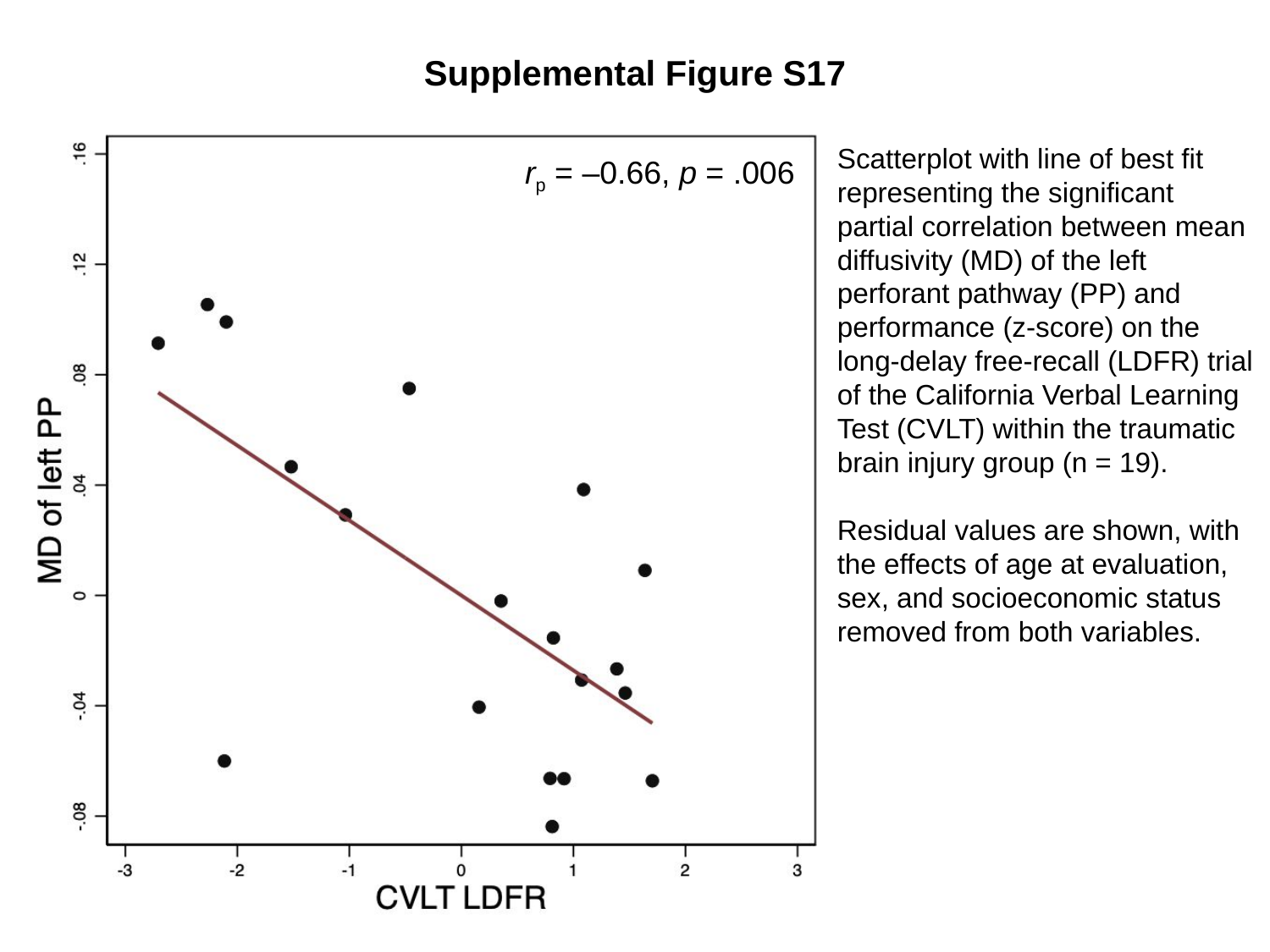

Supplemental Figure S17
Scatterplot with line of best fit representing the significant partial correlation between mean diffusivity (MD) of the left perforant pathway (PP) and performance (z-score) on the long-delay free-recall (LDFR) trial of the California Verbal Learning Test (CVLT) within the traumatic brain injury group (n = 19).
Residual values are shown, with the effects of age at evaluation, sex, and socioeconomic status removed from both variables.
rp = –0.66, p = .006
